# Phylogeny of the spider mite sub-family Tetranychinae (Acari: Tetranychidae) inferred from RNA-Seq data

**DOI:** 10.1371/journal.pone.0203136

**Published:** 2018-09-07

**Authors:** Tomoko Matsuda, Toshinori Kozaki, Kazuo Ishii, Tetsuo Gotoh

**Affiliations:** 1 Faculty of Agriculture, Ibaraki University, Ami, Ibaraki, Japan; 2 Faculty of Agriculture, Tokyo University of Agriculture & Technology, Fuchu, Tokyo, Japan; Nanjing Agricultural University, CHINA

## Abstract

Phylogenetic trees of spider mites were previously obtained using 18S and 28S rRNA genes. Because some of the bootstrap values were relatively low, these trees were unable to completely resolve the phylogeny. Here, we obtained RNA-Seq data for the 72 known species (73 strains) of spider mites to analyze the phylogeny of the sub-family Tetranychinae. The data were *de novo* assembled into a total alignment length of 790,047 bases corresponding to 264,133 amino acid residues in 652 genes. The sequence dataset was 200 times larger than the data used in the previous study. The new trees were much more robust and more clearly defined the clades of the tribes and the genera of the sub-family Tetranychinae. The tribe Tetranychini was polyphyletic because a monophyletic clade of Eurytetranychini was placed inside it. The six genera from which two or more species were sampled appeared to be monophyletic, but four genera (*Schizotetranychus*, *Eotetranychus*, *Oligonychus* and *Tetranychus*) appeared to be polyphyletic. These results strongly support the previous molecular inference of the polyphyletic tribes and genera, although the molecular phylogeny of the sub-family Tetranychinae does not fully agree with the current morphology-based taxonomy. The taxonomy of the sub-family Tetranychinae should be revised according to the molecular relationships revealed by this study.

## Introduction

Phytophagous spider mites (family Tetranychidae) consist of two sub-families (Bryobiinae and Tetranychinae), six tribes (Bryobiini, Hystrichonychini, Petrobiini, Eurytetranychini, Tenuipalpoidini and Tetranychini), 84 genera and more than 1,300 species [1]. The genera of the family Tetranychidae have various feeding habits [2]. For example, *Tetranychus urticae* Koch, *Panonychus citri* (McGregor) and *Oligonychus coffeae* (Nietner) being notorious pests in agriculture have a wide range of host plants. However, the genera *Tetranychus*, *Panonychus* and *Oligonychus* also include monophagous and oligophagous species. For example, *Tetranychus bambusae* Wang & Ma, *Panonychus bambusicola* Ehara & Gotoh, *Oligonychus orthius* Rimando and *Oligonychus rubicundus* Ehara inhabit mainly gramineous plants. Previous phylogenetic studies based on molecular data suggested that phylogenetic relationships of some genera and species inhabiting specific plants are closely linked with their feeding habit [3, 4].

The molecular phylogeny of the sub-family Tetranychinae was first based on the cytochrome *c* oxidase subunit I (COI) gene of the mitochondrial DNA [4, 5] and then on the internal transcribed spacer 2 (ITS2) region of the nuclear ribosomal RNA (rRNA) [6]. The phylogeny was not well resolved in those studies because of the low bootstrap values for most of the nodes, but it showed that the genus *Oligonychus* was apparently polyphyletic. An analysis based on 18S and 28S rRNA [3] confirmed the polyphyly of the genus *Oligonychus* with high bootstrap values. Four *Oligonychus* species whose aedeagi curved dorsally formed a clade with 21 *Tetranychus* species whose aedeagi also curved dorsally. This clade was well separated from 12 other *Oligonychus* species whose aedeagi curved ventrally. In addition, the other three genera, *Tetranychus*, *Schizotetranychus* and *Eotetranychus*, turned out to be polyphyletic [3]. The molecular phylogeny of the sub-family Tetranychinae did not agree with the current taxonomy, but the resolution and the reliability of the phylogenetic trees were not enough to resolve the discrepancy.

RNA-Seq using next generation sequencing is a cost effective method for obtaining orthologous genes for phylogenetic analysis and has greatly improved phylogenetic studies of non-model taxa [7–9]. In species of the malaria mosquito (*Anopheles*), phylogenies obtained with 533 (≥100 bp) and 69 protein-coding genes (≥300 bp) gave much better resolution than previous phylogenic analyses based on a few loci [10]. In the arachnid order Opiliones, 300 protein-coding genes supported a classical hypothesis of the phylogeny, and revealed that the origin of Opiliones was deeper than that indicated by the fossil record [7]. In addition, phylogenetic analysis of the order Lepidoptera with 2,212 protein-coding genes of 28 species considerably improved the bootstrap values compared to those of previous PCR-based analyses [11].

Previous phylogenetic studies of the sub-family Tetranychinae by ourselves and other groups have used mitochondrial DNA or nuclear ribosomal RNA [3–6], but nuclear protein-coding genes have never been used. We previously proposed that analysis of a large number of protein-coding genes would help to resolve the phylogenetic positions of the genus *Eotetranychus* and *Stigmaeopsis*, which could not be elucidated by the 18S and 28S rRNA genes [3]. In this study, RNA-Seq was performed on 72 species (73 strains) of spider mites. Then, 652 protein-coding genes that were orthologous among the 73 strains were collected from the *de novo* assemblies of these spider mites for phylogenic analysis. Our results confirmed the phylogeny of the sub-family Tetranychinae with high bootstrap supports on each of the clades of the tribes and the genera.

## Materials and methods

### Spider mites

The spider mite family Tetranychidae comprises 2 sub-families (Tetranychinae and Bryobiinae) and 6 tribes [1]. We obtained 72 species belonging to 4 tribes, but could not obtain species belonging to two tribes, Hystrichonychini (Bryobiinae) and Tenuipalpoidini (Tetranychinae). Hystrichonychini, comprising 21 genera and more than 160 species in the world [1], is represented with only one species in Japan (*Tetranycopsis borealis* Ehara & Mori) [12], which is difficult to obtain. While the tribe Tetranychini alone comprises more than half of the members of the family Tetranychidae worldwide, Tenuipalpoidini have only 14 known species [1] and none have been described from Japan. In total 72 species (73 strains) of spider mites were used, covering the 2 sub-families Tetranychinae (2 tribes, 11 genera, 68 species, 69 strains) and the Bryobiinae as outgroup (2 tribes, 3 genera, 4 species, 4 strains) (Table 1).

**Table 1:** Classification and sources of tetranychid mites used in this study.

Sub-family	Tribe	Genus	Speces	Date	Locality	Host plant	Voucher specimen no.^†^
Bryobiinae	Bryobiini	*Bryobia*	*B*. *eharai* Pritchard & Keifer	Sept. 11, 2012	Ibaraki, Japan	*Chrysanthemum morifolium*	0612
			*B*. *praetiosa* Koch	July 27, 2008	Hokkaido, Japan	*Trifolium repens*	0609
	Petrobiini	*Petrobia*	*Pe*. *latens* (Müller)	Mar. 30, 2012	Tokushima, Japan	*Daucus carota*	0482
		*Tetranychina*	*Tetranychina harti* (Ewing)	June 11, 2012	Ibaraki, Japan	*Oxalis corniculata*	0602
Tetranychinae	Eurytetranychini	*Eutetranychus*	*Eu*. *africanus* (Tucker)	June 30, 2008	Taichung, Taiwan	*Pueraria montana*	0377
		*Aponychus*	*Ap*. *corpuzae* Rimando	Apr. 10, 2001	Ibaraki, Japan	*Sasa senanensis*	0607
			*Ap*. *firmianae* (Ma & Yuan)	Sept. 11, 2012	Ibaraki, Japan	*Firmiana simplex*	0604
	Tetranychini	*Panonychus*	*Pa*. *bambusicola* Ehara & Gotoh	June 4, 1989	Hokkaido, Japan	*Sasa senanensis*	0606
			*Pa*. *caglei* Mellot	Mar. 18, 2010	Okinawa, Japan	*Trichosanthes pilosa*	0608
			*Pa*. *citri* (McGregor)	May 6, 1993	Ibaraki, Japan	*Ilex crenata*	0226
			*Pa*. *mori* Yokoyama	Apr. 22, 2007	Hokkaido, Japan	*Morus australis*	0239
			*Pa*. *osmanthi* Ehara & Gotoh	June 15, 2010	Tokyo, Japan	*Osmanthus* sp.	0600
			*Pa*. *thelytokus* Ehara & Gotoh	Aug. 1, 2012	Hokkaido, Japan	*Ulmus davidiana*	0584
			*Pa*. *ulmi* (Koch)	Aug. 2, 2012	Nagano, Japan	*Malus pumila*	0603
		*Sasanychus*	*Sa*. *akitanus* (Ehara)	June 23, 1986	Hokkaido, Japan	*Sasa senanensis*	0605
			*Sa*. *pusillus* Ehara & Gotoh	July 31, 2012	Hokkaido, Japan	*Sasa chartacea*	0575
		*Schizotetranychus*	*Sc*. *bambusae* Reck	Sept. 21, 2010	Tokyo, Japan	*Chimonobambusa marmorea*	0536
			*Sc*. *cercidiphylli* Ehara	Sept. 10, 2014	Hokkaido, Japan	*Cercidiphyllum japonicum*	0659
			*Sc*. *gilvus* Ehara & Ohashi	May 22, 2012	Nara, Japan	*Quercus gilva*	0549
			*Sc*. *lespedezae* Begljarov & Mitrofanov	Sept. 1, 2012	Ibaraki, Japan	*Pueraria montana*	0561
			*Sc*. *recki* Ehara	Aug. 4, 2010	Hokkaido, Japan	*Sasa senanensis*	0408
			*Sc*. *schizopus* (Zacher)	Aug. 30, 2012	Ibaraki, Japan	*Salix integra*	0637
			*Sc*. *shii* (Ehara)	May 11, 2011	Ibaraki, Japan	*Castanopsis sieboldii*	0511
		*Stigmaeopsis*	*St*. *celarius* Banks	Aug. 7, 2011	Ibaraki, Japan	*Pleioblastus chino*	0506
			*St*. *longus* (Saito)	June 4, 1989	Hokkaido, Japan	*Sasa senanensis*	0542
			*St*. *miscanthi* (Saito)	June 4, 2014	Chiba, Japan	*Miscanthus sinensis*	0863
			*St*. *saharai* Saito & Mori	June. 4, 2014	Chiba, Japan	*Pleioblastus chino*	0650
			*St*. *takahashii* Saito & Mori	Oct. 27, 1997	Hokkaido, Japan	*Sasa senanensis*	0541
		*Yezonychus*	*Y*. *sapporensis* Ehara	May 11, 2011	Ibaraki, Japan	*Sasa senanensis*	0510
		*Eotetranychus*	*Eo*. *asiaticus* Ehara	Mar. 19, 2007	Nagasaki, Japan	*Citrus reticulata*	0546
			*Eo*. *dissectus* Ehara	July 2, 2014	Hokkaido, Japan	*Acer pictum*	0674
			*Eo*. *nomurai* Ehara	Sept. 9, 2014	Ibaraki, Japan	*Celtis sinensis*	0660
			*Eo*. *pruni* (Oudemans)	July 30, 2014	Gumma, Japan	*Corylus sieboldiana*	0657
			*Eo*. *querci* Reeves	Sept. 10, 2014	Hokkaido, Japan	*Tilia japonica*	0673
			*Eo*. *rubricans* Ehara	Sept. 1, 2012	Ibaraki, Japan	*Carpinus tschonoskii*	0559
			*Eo*. *smithi* Pritchard & Baker	Aug. 14, 2007	Nagasaki, Japan	*Rosa multiflora*	0545
			*Eo*. *suginamensis* (Yokoyama)	June 12, 2012	Tokyo, Japan	*Morus australis*	0601
			*Eo*. *tiliaecola* Ehara & Gotoh	Sept. 10, 2014	Hokkaido, Japan	*Tilia maximowicziana*	0675
			*Eo*. *toyoshimai* Ehara & Gotoh	Sept. 9, 2014	Ibaraki, Japan	*Magnolia obovata*	0651
			*Eo*. *uchidai* Ehara	July 31, 2012	Hokkaido, Japan	*Ulmus davidiana*	0578
			*Eo*. *uncatus* Garman	July 30, 2014	Gumma, Japan	*Betula ermanii*	0656
		*Oligonychus*	*O*. *amiensis* Ehara & Gotoh	July 13, 2005	Ibaraki, Japan	*Lithocarpus edulis*	0116
			*O*. *biharensis* (Hirst)	June 30, 2008	Taipei, Taiwan	*Dimocarpus longan*	0064
			*O*. *camelliae* Ehara & Gotoh	May 13, 2000	Fukushima, Japan	*Camellia japonica*	0082
			*O*. *coffeae* (Nietner)	June 13, 2008	Okinawa, Japan	*Litchi chinensis*	0025
			*O*. *gotohi* Ehara	July 30, 2000	Chiba, Japan	*Lithocarpus edulis*	0096
			*O*. *hondoensis* (Ehara)	Sept. 4, 2014	Ibaraki, Japan	*Cryptomeria japonica*	0652
			*O*. *ilicis* (McGregor)	Oct. 30, 2000	Kagoshima, Japan	*Camellia sinensis*	0081
			*O*. *orthius* Rimando	July 9, 2009	Okinawa, Japan	*Saccharum officinarum*	0378
			*O*. *rubicundus* Ehara	June 15, 2010	Tokyo, Japan	*Miscanthus sinensis*	0599
		*Amphitetranychus*	*Am*. *quercivorus* (Ehara & Gotoh)	July 9, 2003	Ibaraki, Japan	*Quercus crispula*	0610
			*Am*. *viennensis* (Zacher)	May 11, 2007	Ibaraki, Japan	*Cerasus* sp.	0147
		*Tetranychus*	*T*. *bambusae* Wang & Ma	July 5, 2009	Okinawa, Japan	*Phyllostachys edulis*	0343
			*T*. *evansi* Baker & Pritchard	Sept. 21, 2010	Tokyo, Japan	*Solanum nigrum*	0550
			*T*. *ezoensis* Ehara	Sept. 3, 2008	Ibaraki, Japan	*Taxus cuspidata*	0281
			*T*. *huhhotensis* Ehara, Gotoh & Hong	July 26, 2007	Inner Mongolia Autonomous Region, China	*Zea mays*	0201
			*T*. *kanzawai* Kishida	May 19, 1993	Shizuoka, Japan	*Thea sinensis*	0158
			*T*. *lombardinii* Baker & Pritchard	July 10, 2008	Durban, South Africa	*Erythrina variegata*	0381
			*T*. *ludeni* Zacher	Oct.17, 1995	Ibaraki, Japan	*Solidago virgaurea*	0189
			*T*. *macfarlanei* Baker & Pritchard	Sept. 30, 2008	Mymensingh, Bangladesh	*Dolichos lablab*	0389
			*T*. *merganser* Boudreaux	Apr. 6, 2007	Sonora, Mexico	*Cucurbita maxima*	0225
			*T*. *misumaiensis* Ehara & Gotoh	Aug. 23, 2005	Hokkaido, Japan	*Apios* sp.	0218
			*T*. *neocaledonicus* Andre	May 27, 1998	Tokyo, Japan	*Morus australis*	0192
			*T*. *okinawanus* Ehara	June 10, 2008	Okinawa, Japan	*Solanum melongena*	0481
			*T*. *parakanzawai* Ehara	Aug. 16, 2009	Chiba, Japan	*Morus australis*	0339
			*T*. *phaselus* Ehara	June 29, 2000	Ibaraki, Japan	*Glycine max*	0191
			*T*. *piercei* McGregor	Dec. 20, 2007	Okinawa, Japan	*Cucumis melo*	0014
			*T*. *pueraricola* Ehara & Gotoh	Oct. 23, 1993	Ibaraki, Japan	*Pueraria montana*	0203
			*T*. *truncatus* Ehara	May 8, 2004	Kyoto, Japan	*Solanum nigrum*	0195
			*T*. *turkestani* Ugarov & Nikolski	Sept. 15, 2007	Hamedan, Iran	*Phaseolus vulgaris*	0219
			*T*. *urticae* Koch (green form)	Feb. 20, 2006	Ibaraki, Japan	*Fragaria × ananassa*	0185
			*T*. *urticae* Koch (red form)	Aug. 27, 2001	Nagano, Japan	*Dianthus* sp.	0171
			*T*. *zeae* Ehara, Gotoh & Hong	July 26, 2007	Inner Mongolia Autonomous Region, China	*Zea mays*	0202

^†^ Voucher specimens were preserved at the Laboratory of Applied Entomology and Zoology, Faculty of Agriculture, Ibaraki University.

For *T*. *urticae*, 2 strains, a green form: vs# 0185 and a red form: vs# 0171, were used. Among the mite strains, those that could be reared in the laboratory were maintained on the leaves of the common bean *Phaseolus vulgaris* L., the mulberry *Morus bombycis* Koidz., or of the original host plants as described previously [3]. Strains that could not be maintained in the laboratory were preserved in 70% ethanol for morphological identification. Specimens were mounted in the Hoyer’s medium and identified under phase-contrast and differential interference-contrast microscopes. Voucher specimens were prepared as described previously [3] and were preserved in the Laboratory of Applied Entomology and Zoology, Faculty of Agriculture, Ibaraki University.

### Sequencing and *de novo* assembly

Total RNA was prepared by NucleoSpin^®^ RNA XS (Macherey-Nagel, Düren, Germany). Total RNA of the mite strains that were reared in the laboratory was extracted from whole bodies of 100–200 adult females of same population, which were maintained on the same leaf discs (S1 Table). For strains that could not be maintained in the laboratory, total RNA was extracted from whole bodies of 100–200 adult females as soon as they were collected from single plant individuals (S1 Table). Live female individuals for RNA samples and female individuals for voucher specimen were obtained from the same leaf discs and plants. The quantity and quality of the total RNA were evaluated by RNA 6000 nano chips on Agilent 2100 Bioanalyzer (Agilent Technologies, Santa Clara, CA, USA). The cDNA libraries were prepared from the total RNA with a TruSeq RNA sample prep kit (Illumina, San Diego, CA, USA), and the single ends were sequenced for 100 cycles on HiSeq2000 (Illumina). All the reads were deposited in DDBJ Sequence Read Archive (accession number: DRA007145). The sequence reads were trimmed by fastx_trimmer of the FASTX-Toolkit [13] with a parameter -f 15 and by fastq_quality_trimmer with parameters -t 28 and -l 40, and then were filtered by fastq_quality_filter with parameters -q 28 and -p 80. The processed sequence reads were assembled per strain by VELVET [14] and OASES [15] with *k*-mer 51.

### Identification of the orthologs

Contigs with 95% or more similarity were judged to be redundant and were removed from the 73 assemblies by CD-HIT [16]. The open-reading frames (ORFs) were identified by TransDecoder [17]. The contigs were annotated by TBLASTX with a cut-off E-value of 1x10^-50^ against the coding sequences (CDS) from the *T*. *urticae* genome (https://bioinformatics.psb.ugent.be/gdb/tetranychus/mRNA_pseudo_tetur__cds_20150904.tfa) [18]. The orthologous gene was then checked in reciprocal TBLASTX searches where the CDSs of *T*. *urticae* were used as queries against the contigs of the 73 assemblies. If one CDS was paired with two or more contigs of an assembly, the top hit was taken as the contig of the orthologous gene. Then, 1,177 genes were identified as putative ortholog.

Sets of orthologous with identical annotations from each of the 73 assemblies were aligned by DIALIGN-TX [19] with L option to get the longest open reading frame. Poorly aligned regions were removed by the automated option of pgtrimal in Phylogears2 [20]. The aligned sequences were translated into amino acid sequences, which were then re-aligned using MAFFT [21]. Poorly aligned regions were removed using the ‘automated’ option of the Trimal [22]. Of these 1,177 genes, 443 were discarded because the alignments of their amino acid sequences had either less than 100 amino acid residues and/or large gaps (accounting for more than 90% of the positions in the alignments). Then, phylogenetic trees based on each gene individually were constructed using RAxML [23]. We examined the phylogenetic trees based on each orthologous gene visually and removed obvious paralogous genes. After removal of these genes including suspected paralogous sequences, 652 putative orthologous genes (total alignment length = 790,047 bases or 264,133 amino acid residues) (S2 Table) remained and were used for the following phylogenetic analyses.

Four species (*Panonychus thelytokus* Ehara & Gotoh: vs# 0584, *Schizotetranychus gilvus* Ehara & Ohashi: vs# 0549, *Oligonychus gotohi* Ehara: vs# 0096, *Oligonychus hondoensis* (Ehara): vs# 0652) had more gaps in the nucleotide and amino acid alignments than other species due to poor assembly of sequence reads as shown by their lower N50 values (S1 Table). For three of these species (all but *O*. *gotohi*), total RNA was extracted immediately after collection in the field. Although *Pa*. *thelytokus*, *Sc*. *gilvus* and *O*. *hondoensis* were collected from single plants, their genetic diversities could be higher than those of strains that have been reared in the laboratory for long periods. However, we did not remove these three species or *O*. *gotohi* from the phylogenetic analyses, because several studies using empirical and simulated data (e.g., [24–27]) have shown that taxa with extensive missing data could be accurately placed in phylogenetic analyses without significantly affecting the results. Indeed, the topology of a tree based on the nucleotide sequences excluding these four species (S1 Fig) did not conflict with the tree constructed with the complete alignments (see Results).

### Phylogenic analysis

The alignments of orthologous genes were concatenated into the combined dataset for the phylogenetic analyses. We constructed two datasets for phylogenetic analyses: (i) nucleotide dataset of all orthologous genes (652 genes, total alignment length = 790,047 bases); and (ii) amino acid dataset of all orthologous genes (652 genes, total alignment length = 264,133 amino acid residues). For the nucleotide datasets, we used the GTRGAMMA model and conducted gene- and codon-partitioned maximum likelihood analyses using RAxML [23]. For the amino acid datasets, we chose the best fitting model for each gene with "ProteinModelSelection.pl" script available with the RAxML and then conducted gene-partitioned maximum-likelihood analyses using RAxML. All RAxML searches were executed for the best-scoring ML tree in one single run (using the ‘-f a’ option). Statistical support values were evaluated with 100 rapid bootstrap inferences.

## Results

### Sequencing, *de novo* assembly and extraction of orthologous genes

The median number of sequence reads for the 73 strains was 16.3M (S1 Table). The reads were quality-filtered and *de novo* assembled into contigs. Then, 1,177 putative orthologous genes were identified using reciprocal TBLASTX searches against coding DNA sequences (CDSs) of *T*. *urticae* [18]. Each putative orthologous gene was aligned individually and used to construct phylogenetic trees. The trees based on each gene were examined visually and obvious paralogous genes were excluded. After these exclusions, 652 putative orthologous genes (S2 Table, total alignment length = 790,047 bases or 264,133 amino acid residues) remained and were used for the following phylogenetic analyses.

### Phylogenetic trees of the sub-family Tetranychinae

**Fig 1:**
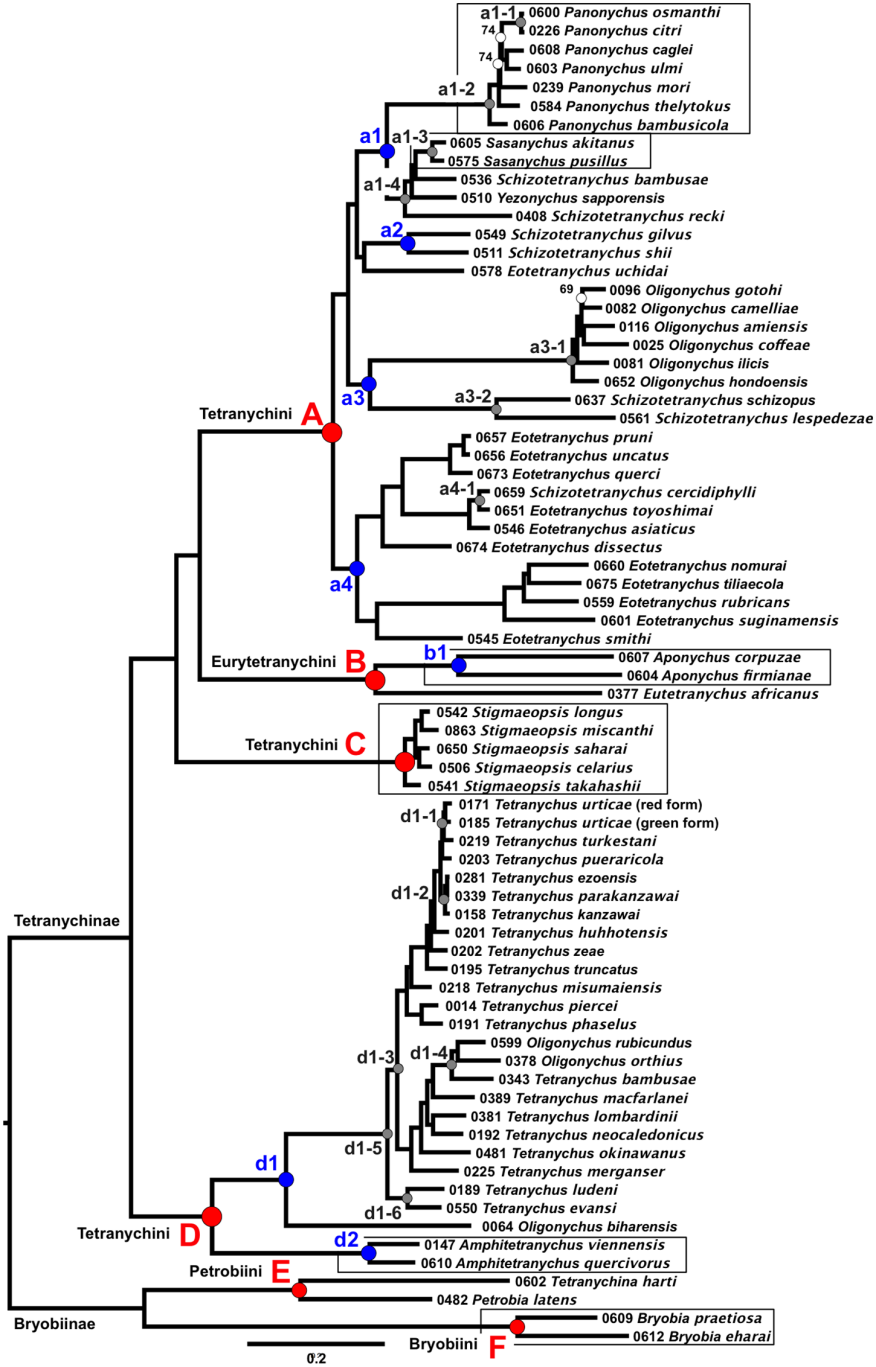
Maximum likelihood (ML) phylogenetic tree of the sub-family Tetranychinae based on the nucleotide sequences (73 operational taxonomic unit (OTU), 652 genes, total alignment length = 790,047 bases). Each OTU is indicated by the voucher specimen no. and scientific name. White dots indicate nodes that are not supported by bootstrap values of 100%. The coded red, blue and gray dots indicate clade nos. which correspond with the clades mentioned in the running text. The red dots indicate clades that represent species belonging to the same tribe, the blue and gray dots indicate sub-clades of red and blue, respectively. The boxes indicate genera that appear monophyletic.

**Fig 2:**
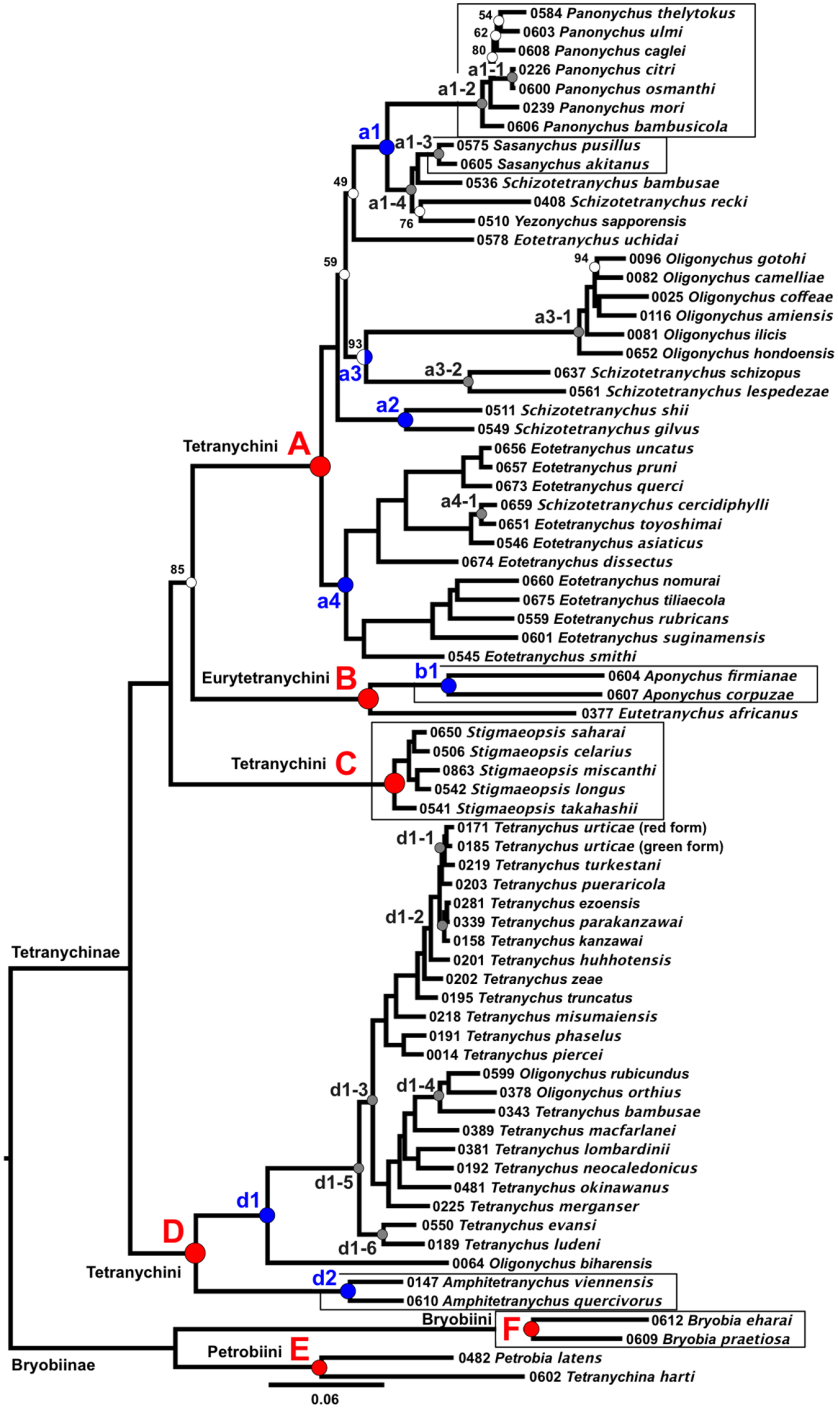
Maximum likelihood (ML) phylogenetic tree of the sub-family Tetranychinae based on the amino acid sequences (73 operational taxonomic unit (OTU), 652 genes, total alignment length = 264,133 amino acid residues). Each OTU is indicated by the voucher specimen no. and scientific name. White dots indicate nodes that are not supported by bootstrap values of 100%. The coded red, blue and gray dots indicate clade nos. which correspond with the clades mentioned in the running text. The red dots indicate clades that represent species belonging to the same tribe, the blue and gray dots indicate sub-clades of red and blue, respectively. The boxes indicate genera that appear monophyletic.

Phylogenic trees were constructed based on the nucleotide sequences (Fig 1) and the amino acid sequences (Fig 2). Most of the nodes in the two trees were highly supported, with 100% bootstrap values in 67 of the 70 nodes in Fig 1 and in 61 of the 70 nodes in Fig 2. The total length of the nucleotide tree (9.43) was three times greater than that of the amino acid tree (3.49).

**Fig 3:**
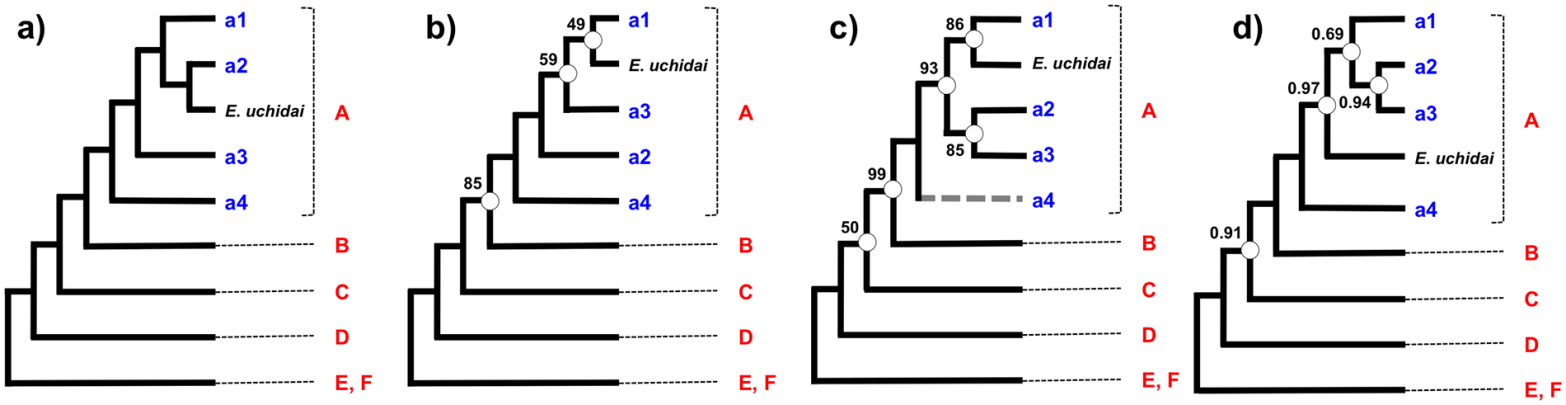
Schematic phylogeny of the spider mites. Except for *Eo*. *uchidai*, each operational taxonomic unit (OTU) is indicated by a symbol corresponding to Figs 1 and 2 (A-F and a1-a4). White dots indicate nodes that are not supported by bootstrap values of 100% or posterior probabilities of 1.0. a) Maximum likelihood (ML) tree based on the nucleotide sequences (Fig 1, 652 genes, total alignment length = 790,047 bases). b) ML tree based on the amino acid sequences (Fig 2, 652 genes, total alignment length = 264,133 amino acid residues). c) ML tree of the 18S and 28S rRNA genes [3]. Branch with the dotted gray line indicates that *Eotetranychus* species and *Sc*. *cercidiphylli* in this branch was not monophyletic but paraphyletic. d) Bayesian tree of the 18S and 28S rRNA genes [3].

The sub-family Bryobiinae was used as an outgroup. Two tribes of the Briobiinae (Petrobiini (clade E) and Bryobiini (clade F)) were monophyletic in both trees. In the sub-family Tetranychinae, the tribe Eurytetranychini (clade B) was monophyletic but was included in a clade with the tribe Tetranychini (Figs 1 and 2, clades A, C and D). At the genus level, genera that were monophyletic included *Bryobia* (clade F), *Aponychus* (clade b1), *Sasanychus* (clade a1-3), *Panonychus* (clade a1-2), *Stigmaeopsis* (clade C) and *Amphitetranychus* (clade d2), whereas genera that were polyphyletic included *Schizotetranychus*, *Eotetranychus*, *Oligonychus* and *Tetranychus*. These results coincided with previous phylogenic analyses based on the 18S and 28S rRNA genes (Fig 3) [3].

The nucleotide and the amino acid trees showed the same topology with 3 exceptions: (i) *Sc*. *gilvus*: vs# 0549 and *Schizotetranychus shii* (Ehara): vs# 0511 formed a clade with *Eotetranychus uchidai* Ehara: vs# 0578 in the nucleotide tree (Fig 1, clade a2), but not in the amino acid tree (Fig 2); (ii) *Schizotetranychus recki* Ehara: vs# 0408 was located at the root of the clade including *Sasanychus akitanus* (Ehara): vs# 0605, *Sasanychus pusillus* Ehara & Gotoh: vs# 0575, *Schizotetranychus bambusae* Reck: vs# 0536 and *Yezonychus sapporensis* Ehara: vs# 0510 in the nucleotide tree, but not in the amino acid tree (Figs 1 and 2, clade a1-4); (iii) the topology of the clade of the genus *Panonychus* was different in the two trees and was ambiguous, because the bootstrap values for some nodes were relatively low (Figs 1 and 2, clade a1-2).

The *Schizotetranychus* species were scattered across clade A (Figs 1 and 2), but they formed four well-supported clades: (i) *Sc*. *bambusae*: vs# 0536 and *Sc*. *recki*: vs# 0408 clustered with the *Sasanychus* and the *Yezonychus* species (Figs 1 and 2, clade a1-4); (ii) *Sc*. *gilvus*: vs# 0549 and *Sc*. *shii*: vs# 0511 formed a cluster (Figs 1 and 2, clade a2); (iii) *Schizotetranychus lespedezae* Begljarov & Mitrofanov: vs# 0561 and *Schizotetranychus schizopus* (Zacher): vs# 0637 formed a cluster (Figs 1 and 2, clade a3-2); (iv) *Schizotetranychus cercidiphylli* Ehara: vs# 0659 was located in an *Eotetranychus* clade (Figs 1 and 2, clade a4), and clustered with *Eotetranychus toyoshimai* Ehara & Gotoh: vs# 0651 (Figs 1 and 2, clade a4-1).

Species of the genus *Oligonychus* are separated into 2 clades (Figs 1 and 2, clades A and D). Clade A includes *Oligonychus* species whose aedeagi curve ventrally (Figs 1 and 2, clade a3-1) and clade D includes *Oligonychus* species whose aedeagi curve dorsally and *Amphitetranychus* and *Tetranychus* species, all of which also have dorsally curved aedeagi. In clade D, *Amphitetranychus* was monophyletic (Figs 1 and 2, clade d2), but *Oligonychus* and *Tetranychus* were polyphyletic (Figs 1 and 2, clade d1). *Oligonychus* species were scattered across clade D in two groups: (i) *Oligonychus biharensis* (Hirst): vs# 0064 was located at the root of the clade including other *Oligonychus* and *Tetranychus* species (Figs 1 and 2, clade d1-5); (ii) *O*. *rubicundus*: vs# 0599 and *O*. *orthius*: vs# 0378 clustered with *T*. *bambusae*: vs# 0343 (Figs 1 and 2, clade d1-4).

## Discussion

The RNA-Seq datasets used for the phylogenetic analyses of this study (652 genes, total alignment length = 790,047 bases or 264,133 amino acid residues) were significantly larger than the dataset of the previous study based on the 18S and 28S rRNA (2 genes, total alignment length = 2,534 bases) [3]. This study provides a mostly well-resolved and robustly supported phylogeny of the sub-family Tetranychinae. The most compelling results of this study are that almost all the nodes were supported by bootstrap values of 100% (Figs 1 and 2) and most topology was consistent with the previous studies (Fig 3). In addition, four associations between spider mites and their host plants found in the previous study were confirmed in the present study: (i) *Oligonychus* and *Tetranychus* species inhabiting gramineous plants (*O*. *rubicundus*: vs# 0599, *O*. *orthius*: vs# 0378 and *T*. *bambusae*: vs# 0343) clustered separately from other species and formed a monophyletic clade (Figs 1 and 2, clade d1-4); (ii) clade a1-4 (Figs 1 and 2) includes species of three genera that inhabit gramineous plants: two *Sasanychus* species (*Sa*. *akitanus*: vs# 0605 and *Sa*. *pusillus*: vs# 0575), two *Schizotetranychus* species (*Sc*. *recki*: vs# 0408 and *Sc*. *bambusae*: vs# 0536) and one *Yezonychus* species (*Y*. *sapporensis*: vs# 0510); (iii) all *Stigmaeopsis* species inhabiting gramineous plants are separated from other Tetranychini species and form a monophyletic (clade C); (iv) clade a2 (Figs 1 and 2) includes *Sc*. *gilvus*: vs# 0549 and *Sc*. *shii*: vs# 0511 which inhabit fagaceous plants. These results demonstrate that RNA-Seq analyses are useful for inferring the phylogeny of the spider mite sub-family Tetranychinae.

The main purpose of this study was to resolve the phylogeny of the sub-family Tetranychinae, especially, the phylogenetic positions of the genera *Stigmaeopsis* and *Eotetranychus*, which could not be elucidated by the 18S and 28S rRNA genes [3]. In this study, the genus *Stigmaeopsis* formed a well-supported clade (clade C) in both trees (Figs 1 and 2). Also in both trees, clade C (Tetranychini) clustered with clade A (Tetranychini) and clade B (Eurytetranychini) with 100% bootstrap values. These relationships are summarized in Fig 3. *Eotetranychus* species, with the exception of *Eo*. *uchidai*: vs# 0578, formed a well-supported clade (bootstrap value = 100%) with *Sc*. *cercidiphylli*: vs# 0659 (Figs 1 and 2, clade a4). *Eo*. *uchidai*, which does not cluster with other *Eotetranychus* species, clustered with *Sc*. *gilvus*: vs# 0549 and *Sc*. *shii*: vs# 0511 (Fig 1, clade a2) in the nucleotide tree. However, in the amino acid tree, the bootstrap values were too low to establish the exact phylogenetic position of *Eo*. *uchidai* (Fig 2, bootstrap value = 49%). Furthermore, the position of *Eo*. *uchidai* is not congruent with its position in previous trees based on the 18S and 28S rRNA genes [3] (Fig 3). Further studies of undescribed *Eotetranychus* species throughout the world and increased gene sampling are needed to resolve the phylogenetic position of *Eo*. *uchidai*.

The topology presented here does not fully agree with the current taxonomy of the spider mites based on morphology. At the tribe level, the tribe Eurytetranychini (clade B) was monophyletic but was included in a clade with the tribe Tetranychini (Figs 1 and 2, clades A, C and D). At the genus level, four genera (*Schizotetranychus*, *Eotetranychus*, *Oligonychus* and *Tetranychus*) were polyphyletic. The present results confirm the discrepancy between the morphological and molecular taxonomies. However, some phylogenetic patterns of spider mites are associated with morphological characters. For example, three *Oligonychus* species (*O*. *rubicundus*: vs# 0599, *O*. *orthius*: vs# 0378 and *O*. *biharensis*: vs# 0064) whose aedeagi curved dorsally were very closely related to *Tetranychus* species whose aedeagi also curved dorsally [12]. Two *Sasanychus* species, which are considered as subgenera of *Panonychus* [2], have been proposed to form an independent genus because the dorsal idiosomal setae do not set on tubercles and the hysterosoma has transverse striae in the dorsocentral area [28]. Our phylogenetic trees show that the genera *Panonychus* and *Sasanychus* are clearly separated into two distinct clades (Figs 1 and 2, clades a1-2 and a1-3) and support the morphological classification proposed by Ehara and Gotoh [28]. At the species level, *Tetranychus evansi* Baker & Pritchard: vs# 0550 and *Tetranychus ludeni* Zacher: vs# 0189, which are similar in the arrangement of setae in the female tarsus I [12], were confirmed to form a monophyletic clade (Figs 1 and 2, clade d1-6) apart from the other *Oligonychus* and *Tetranychus* species (clade d1-3). *Tetranychus kanzawai* Kishida: vs# 0158, *Tetranychus parakanzawai* Ehara: vs# 0339 and *Tetranychus ezoensis* Ehara: vs# 0281, which are morphologically close to each other and which are separated only by the diameter of the aedeagal knobs of the males (4 μm in *T*. *kanzawai*, 3.3 μm in *T*. *parakanzawai* and 3.5 μm in *T*. *ezoensis*) [12], were very close in the phylogenic trees (Figs 1 and 2, clade d1-2). The two forms of *T*. *urticae* (green: vs# 0185 and red: vs# 0171 forms) and *Tetranychus turkestani* Ugarov & Nikolskii: vs# 0219 which are closely related species and are not identifiable in the COI tree [29], were also closely related in our phylogenetic trees (Figs 1 and 2, clade d1-1). *Panonychus osmanthi* Ehara & Gotoh: vs# 0600 morphologically resembles *Pa*. *citri*: vs# 0226 and produced sterile F1 females when mated with *Pa*. *citri* [12, 30, 31]. These two species formed a cluster as described in previous phylogenetic analyses [3, 32] (Figs 1 and 2, clade a1-1). These results confirm that molecular evidence together with morphological characters can clarify the phylogenic relations of spider mites.

## Conclusions

Our results strongly support the previous molecular phylogeny inferred by the 18S and 28S rRNA genes [3], and give high resolution to the phylogenetic positions of the genera *Stigmaeopsis* and *Eotetranychus* and closely related species of spider mites. The clustering of the tribes and genera in the phylogenic trees do not fully agree with the current taxonomy. This inconsistency suggests that the current taxonomy should be reconsidered based on the molecular evidence of this study.

## Supporting information

S1 Table Summary of mite samples, sequencing, *de novo* assembly, filtration of contigs and gaps/missing data in dataset. (PDF)

S2 Table List of genes used for phylogenetic analyses. (PDF)

S1 Fig Maximum likelihood (ML) phylogenetic tree of the sub-family Tetranychinae based on the nucleotide sequences (69 operational taxonomic unit (OTU), 652 genes, total alignment length = 790,047 bases). (PDF)
