# Supplementary material for: Phylogeny of the spider mite sub-family Tetranychinae (Acari: Tetranychidae) inferred from RNA-Seq data
Source: PLoS One. 2018 Sep 7;13(9):e0203136. doi: 10.1371/journal.pone.0203136 (PMC6128517; doi:10.1371/journal.pone.0203136)
Supplement: S1 Table — (PDF) [file pone.0203136.s001.pdf]

S1 Table Summary of mite samples, sequencing, de novo assembly, filtration of contigs and gaps/missing data in dataset

		Mite samples	Sequencing			*de novo* assembly				Filtration of contigs		Gap/missing data in dataset	
Species	Voucher specimen no.	Maintained in the laboratory or collected in the field	Number of reads	% of >= Q30 Bases (passing filter)	Mean Quality Score (passing filter)	Number of contigs	Number of N50 contigs	N50 length	Max length of contigs (bp)	Number of contigs after CD-HIT	Number of contigs after TransDecoder	% of gaps/missing data in nucleotide dataset	% of gaps/missing data in amino acid dataset
B. eharai Pritchard & Keifer	0612	Field	14,272,657	95.38	36.81	18,316	3,258	1,951	12,885	16,401	10,865	5.12	5.66
B. praetiosa Koch	0609	Laboratory	31,336,662	92.36	35.98	19,869	3,530	2,173	18,418	17,179	11,416	3.90	4.30
Pe. latens (Müller)	0482	Field	16,125,279	96.47	37.43	23,623	3,899	1,926	14,249	19,835	11,057	4.89	5.48
Tetranychina harti (Ewing)	0602	Field	18,682,275	95.57	37.02	17,941	3,032	2,035	13,748	16,163	9,922	4.41	4.69
Eu. africanus (Tucker)	0377	Laboratory	26,254,145	92.00	35.76	20,736	3,509	2,341	15,665	18,345	11,457	3.92	4.57
Ap. corpuzae Rimando	0607	Laboratory	18,187,217	96.06	37.11	21,228	3,618	2,152	13,944	19,526	11,541	3.31	3.65
Ap. firmianae (Ma & Yuan)	0604	Field	23,552,652	92.40	35.86	20,146	3,414	2,134	16,154	18,898	11,611	3.51	3.91
Pa. bambusicola Ehara & Gotoh	0606	Laboratory	15,097,372	96.21	37.29	17,816	3,024	1,920	15,642	16,873	10,527	1.63	1.88
Pa. caglei Mellot	0608	Laboratory	22,604,835	96.24	37.33	17,424	3,061	1,697	13,775	16,799	10,429	1.92	2.22
Pa. citri (McGregor)	0226	Laboratory	42,123,202	93.53	36.43	21,104	3,717	2,375	15,226	18,708	12,071	2.09	2.38
Pa. mori Yokoyama	0239	Laboratory	17,817,459	94.66	36.70	18,748	3,143	1,814	12,241	17,777	10,589	1.97	2.31
Pa. osmanthi Ehara & Gotoh	0600	Laboratory	14,717,237	95.29	36.94	18,391	3,183	1,851	13,987	17,402	10,836	2.16	2.55
Pa. thelytokus Ehara & Gotoh	0584	Field	17,298,627	93.55	36.41	12,639	2,248	681	9,434	12,401	5,852	40.87	46.14
Pa. ulmi (Koch)	0603	Field	12,719,324	96.64	37.43	19,590	3,329	1,325	11,535	18,637	10,690	3.39	3.89
Sa. akitanus (Ehara)	0605	Laboratory	14,980,368	95.70	37.11	17,970	2,969	1,920	14,693	17,057	10,502	1.79	1.98
Sa. pusillus Ehara & Gotoh	0575	Field	15,086,380	94.88	36.80	18,304	3,106	1,702	13,847	17,394	10,648	1.43	1.61
Sc. bambusae Reck	0536	Laboratory	15,310,560	96.59	37.46	18,393	3,084	1,833	13,866	17,490	10,618	2.81	3.12
Sc. cercidiphylli Ehara	0659	Field	27,558,733	95.64	36.74	22,056	3,691	1,768	13,348	20,324	11,500	2.86	2.97
Sc. gilvus Ehara & Ohashi	0549	Field	18,618,123	95.01	36.63	19,370	3,060	755	14,659	19,084	8,156	26.87	30.72
Sc. lespedezae Begljarov & Mitrofanov	0561	Field	18,559,733	95.73	36.92	19,433	3,350	1,749	13,277	18,172	11,149	3.17	3.40
Sc. recki Ehara	0408	Laboratory	16,834,420	95.60	37.04	23,748	3,887	1,810	14,530	20,736	11,443	3.03	3.59
Sc. schizopus (Zacher)	0637	Field	18,433,830	92.54	35.94	17,818	3,128	1,842	14,717	16,743	10,461	2.70	3.05
Sc. shii (Ehara)	0511	Field	15,911,589	95.99	37.15	18,086	3,089	1,840	14,372	17,145	10,370	1.66	2.00
St. celarius Banks	0506	Laboratory	14,977,389	95.31	37.02	19,595	3,426	1,977	13,967	18,323	10,955	1.73	1.79
St. longus (Saito)	0542	Laboratory	15,042,975	95.96	37.15	19,456	3,234	1,942	13,886	18,274	10,722	2.21	2.33
St. miscanthi (Saito)	0863	Field	25,639,639	95.42	36.69	20,444	3,504	2,318	16,609	18,650	10,991	1.99	2.01
St. saharai Saito & Mori	0650	Laboratory	17,277,980	96.55	37.16	21,563	3,628	1,572	12,373	20,290	11,226	2.45	2.60
St. takahashii Saito & Mori	0541	Laboratory	23,467,381	93.44	36.34	20,112	3,441	1,968	17,331	18,624	11,086	1.88	1.89
Y. sapporensis Ehara	0510	Laboratory	14,156,455	96.29	37.31	18,714	3,238	1,736	13,278	17,593	10,885	2.71	2.93
Eo. asiaticus Ehara	0546	Laboratory	14,693,257	95.75	37.12	19,059	3,132	1,846	14,557	17,831	10,513	3.01	3.29
Eo. dissectus Ehara	0674	Field	25,074,111	95.29	36.66	20,501	3,050	1,978	21,393	19,405	10,938	2.30	2.49
Eo. nomurai Ehara	0660	Field	16,107,703	96.12	36.95	16,714	2,972	2,095	13,600	15,831	10,644	1.76	2.08
Eo. pruni (Oudemans)	0657	Field	26,475,908	95.38	36.69	20,456	3,275	2,009	21,294	19,296	11,169	2.32	2.49
Eo. querci Reeves	0673	Field	27,041,128	95.08	36.62	22,035	3,680	1,746	19,643	20,457	11,755	2.37	2.44
Eo. rubricans Ehara	0559	Field	24,160,459	93.40	36.24	18,049	3,061	1,707	21,152	16,974	10,607	2.97	3.10
Eo. smithi Pritchard & Baker	0545	Laboratory	33,006,564	96.50	37.21	21,150	3,605	2,465	21,384	19,012	12,403	4.29	4.80
Eo. suginamensis (Yokoyama)	0601	Field	11,059,870	95.07	36.69	17,051	3,090	1,587	8,494	16,477	10,389	3.73	3.95
Eo. tiliaecola Ehara & Gotoh	0675	Field	25,156,846	95.80	36.77	23,782	3,682	1,507	21,501	22,748	12,505	6.13	7.02
Eo. toyoshimai Ehara & Gotoh	0651	Field	26,506,367	94.90	36.58	20,017	3,282	2,314	16,608	18,640	11,689	2.41	2.86
Eo. uchidai Ehara	0578	Field	14,069,875	96.14	37.16	18,028	3,177	1,473	13,176	17,199	10,292	3.28	3.57
Eo. uncatus Garman	0656	Field	28,025,618	95.72	36.76	20,047	3,214	1,797	14,867	18,586	10,485	2.81	3.08
O. amiensis Ehara & Gotoh	0116	Laboratory	16,790,272	95.64	37.31	18,868	3,185	1,077	11,280	18,534	10,045	8.63	9.45
O. biharensis (Hirst)	0064	Laboratory	14,859,172	96.77	37.30	19,997	3,386	1,851	15,851	18,929	11,032	1.94	2.37
O. camelliae Ehara & Gotoh	0082	Laboratory	14,216,115	95.35	37.01	18,158	3,152	1,694	13,083	17,381	10,574	2.33	2.39
O. coffeae (Nietner)	0025	Laboratory	32,166,635	96.14	37.26	19,006	3,155	1,520	13,203	18,417	10,738	2.54	2.60
O. gotohi Ehara	0096	Laboratory	13,879,424	96.54	37.42	17,406	3,009	807	13,191	17,175	8,221	23.20	26.07
O. hondoensis (Ehara)	0652	Field	14,711,796	96.22	36.93	20,585	3,436	990	13,120	19,625	10,073	11.51	12.56
O. ilicis (McGregor)	0081	Field	16,255,963	95.23	37.07	18,831	3,228	1,688	13,162	18,038	10,849	2.03	2.36
O. orthius Rimando	0378	Laboratory	17,609,258	96.74	37.54	21,346	3,515	1,906	14,108	20,040	11,603	1.52	1.91
O. rubicundus Ehara	0599	Laboratory	17,065,767	96.76	37.54	19,209	3,228	1,904	12,395	18,270	10,770	1.54	1.93
Am. quercivorus (Ehara & Gotoh)	0610	Laboratory	14,736,057	95.98	37.20	19,735	3,297	1,923	13,525	18,468	10,544	3.07	3.35
Am. viennensis (Zacher)	0147	Laboratory	15,406,017	95.52	37.00	20,439	3,486	1,630	12,402	19,432	11,140	3.25	3.50
T. bambusae Wang & Ma	0343	Laboratory	18,573,610	95.90	37.24	21,165	3,455	1,955	20,690	20,049	11,616	2.17	2.56
T. evansi Baker & Pritchard	0550	Laboratory	35,858,245	94.56	36.85	25,020	4,355	2,347	21,058	22,432	13,085	3.61	4.13
T. ezoensis Ehara	0281	Laboratory	15,963,830	95.88	37.33	22,765	4,195	1,595	12,215	21,512	12,293	3.22	3.82
T. huhhotensis Ehara, Gotoh & Hong	0201	Laboratory	12,268,481	95.75	37.35	20,525	3,523	1,652	13,062	19,126	11,181	1.82	2.22
T. kanzawai Kishida	0158	Laboratory	31,860,373	94.29	36.74	22,284	3,859	2,025	21,488	20,266	11,846	2.33	2.70
T. lombardinii Baker & Pritchard	0381	Laboratory	13,551,798	96.17	37.49	18,471	3,142	1,626	13,084	17,714	10,630	2.69	3.20
T. ludeni Zacher	0189	Laboratory	17,699,565	96.54	37.46	19,756	3,387	1,834	13,070	18,658	11,088	1.81	2.09
T. macfarlanei Baker & Pritchard	0389	Laboratory	13,039,598	95.92	37.33	18,904	3,299	1,725	12,344	17,991	10,637	2.28	2.97
T. merganser Boudreaux	0225	Laboratory	11,832,575	95.74	37.28	18,211	3,119	1,628	11,418	17,462	10,309	2.64	3.14
T. misumaiensis Ehara & Gotoh	0218	Laboratory	11,869,756	95.73	37.27	18,747	3,244	1,618	10,218	17,951	10,690	1.83	2.13
T. neocaledonicus Andre	0192	Laboratory	16,311,908	95.95	37.30	18,993	3,275	1,736	11,313	18,114	10,882	1.81	2.23
T. okinawanus Ehara	0481	Laboratory	19,862,150	96.17	37.34	23,244	3,815	2,014	14,574	21,731	11,918	2.12	2.61
T. parakanzawai Ehara	0339	Laboratory	18,997,047	95.87	37.28	19,219	3,357	1,604	9,595	18,325	10,974	2.16	2.56
T. phaselus Ehara	0191	Laboratory	15,191,670	95.64	37.33	18,977	3,240	1,754	13,142	18,093	10,898	1.95	2.33
T. piercei McGregor	0014	Laboratory	14,674,987	95.91	37.32	18,312	3,134	1,675	12,992	17,284	10,397	2.33	2.80
T. pueraricola Ehara & Gotoh	0203	Laboratory	11,636,034	96.06	37.38	19,187	3,318	1,388	11,352	18,588	10,642	3.13	3.91
T. truncatus Ehara	0195	Laboratory	16,370,292	96.24	37.46	18,711	3,182	1,663	20,172	18,022	10,601	1.68	2.12
T. turkestani Ugarov & Nikolski	0219	Laboratory	13,721,827	96.04	37.42	21,381	3,648	1,542	10,662	19,520	11,263	3.32	4.03
T. urticae Koch (green form)	0185	Laboratory	13,515,407	96.19	37.45	20,669	3,619	1,615	8,990	18,880	11,063	2.46	2.93
T. urticae Koch (red form)	0171	Laboratory	14,320,356	96.08	37.32	20,917	3,560	1,560	13,085	19,817	11,688	2.46	2.86
T. zeae Ehara, Gotoh & Hong	0202	Laboratory	12,288,281	95.90	37.38	19,293	3,241	1,529	10,786	18,240	10,627	3.34	4.05

*Minimum length of contigs representing 50% of the assembly.
