# Supplementary material for: Phylogeny of the spider mite sub-family Tetranychinae (Acari: Tetranychidae) inferred from RNA-Seq data
Source: PLoS One. 2018 Sep 7;13(9):e0203136. doi: 10.1371/journal.pone.0203136 (PMC6128517; doi:10.1371/journal.pone.0203136)
Supplement: S2 Table — (PDF) [file pone.0203136.s002.pdf]

S2 Table. List of genes using for phylogenetic analyses

Gene ID*	product
tetur01g00170	phosphoserine_aminotransferase
tetur01g02320	Glutathione_S-transferase,_class_omega
tetur01g02450	pyruvate_dehydrogenase
tetur01g02520	calcium-binding_protein_p22
tetur01g02680	electron_transfer_flavoprotein-ubiquinone_oxidoreductase,_mitochondrial_precursor
tetur01g03080	Importin/Karyopherin_subunit_alpha-7_n/a_importin_subunit_alpha-7
tetur01g03240	phosphatidylethanolamine-binding_protein
tetur01g03260	4-hydroxyphenylpyruvate_dioxygenase_n/a_4-hydroxyphenylpyruvate_dioxygenase
tetur01g03280	28S_ribosomal_protein_S18b,_mitochondrial_precursor
tetur01g03930	Actin-related_protein_2/3_complex_subunit_5
tetur01g04390	Nucleosome_assembly_protein_NAP
tetur01g04460	cytochrome_c_oxidase_subunit_Va
tetur01g04550	Acetoacetyl-CoA_thiolase_1
tetur01g05940	ABC-transporter,_class_H_pABC-02_ABC-transporter,_putative
tetur01g06160	Succinyl-CoA_synthetase-like
tetur01g06320	PREDICTED:_similar_to_GMP_synthase
tetur01g06510	thioredoxin-like_1
tetur01g06890	vacuolar_ATP_synthase_subunit_F_n/a_vacuolar_ATP_synthase_subunit_F
tetur01g06950	SNF4/AMP-activated_protein_kinase_gamma_subunit_n/a_5'-AMP-activated_protein_kinase_subunit_gamma-1
tetur01g06960	Citrate_synthase-like,_core
tetur01g06970	Surfeit_locus_protein_4_homolog
tetur01g07310	Carbamoyl-phosphate_synthase_L_chain_ATP-binding
tetur01g07400	eukaryotic_translation_initiation_factor_2_subunit_2
tetur01g07690	Heat_shock_protein_Hsp70
tetur01g08310	GTP-binding_protein_1
tetur01g08700	DEAD
tetur01g08930	galactosylgalactosylxylosylprotein_3-beta-glucuronosyltransferase_1
tetur01g09380	DNA-directed_RNA_polymerases_I,_II,_and_III_subunit_RPABC3
tetur01g09400	tripeptidyl-peptidase_2
tetur01g09650	transketolase
tetur01g10000	acid_phosphatase-1
tetur01g10010	elongation_of_very_long_chain_fatty_acids_protein_6
tetur01g10040	PREDICTED:_similar_to_CG6416-PF,_isoform_F
tetur01g10340	PREDICTED:_sel-1_suppressor_of_lin-12-like
tetur01g10610	phosphatidylinositol_transfer_protein_alpha_isoform
tetur01g11060	Skb1_methyltransferase
tetur01g11470	isochorismatase_domain-containing_protein_1
tetur01g11900	maternal_embryonic_leucine_zipper_kinase
tetur01g12180	peroxisomal_biogenesis_factor_19_n/a_peroxisomal_biogenesis_factor_19
tetur01g12240	Flavin_reductase
tetur01g12790	Methionine_adenosyl_transferase_gene_a
tetur01g13410	DnaJ
tetur01g13550	Vacuolar_ATP_synthase_21_kDa_proteolipid_subunit
tetur01g13710	multifunctional_protein_ADE2
tetur01g13720	amidophosphoribosyltransferase
tetur01g13800	5'-AMP-activated_protein_kinase_subunit_beta-1
tetur01g14030	ATP_synthase_subunit_gamma,_mitochondrial_precursor
tetur01g14250	NADH_dehydrogenase
tetur01g14290	COP9_signalosome_complex_subunit_3
tetur01g14410	solute_carrier_family_29
tetur01g14590	40S_ribosomal_protein_S3a
tetur01g14630	TAR_DNA-binding_protein_43
tetur01g14710	glutaredoxin-like_protein
tetur01g15060	MIP_family_channel_protein
tetur01g15140	Ecdysone_Receptor
tetur01g15220	small_glutamine-rich_tetratricopeptide_repeat-containing_protein_alpha
tetur01g15410	succinate_dehydrogenase_assembly_factor_2,_mitochondrial_precursor
tetur01g15710	succinate_dehydrogenase_[ubiquinone]_iron-sulfur_subunit_n/a_succinate_dehydrogenase_complex_subunit_B_n/a_succinate_dehydrogenase_complex_subunit_B
tetur01g16220	zinc_finger_protein_n/a_PREDICTED:_similar_to_zinc_finger_protein
tetur02g00220	GM2_activator_protein
tetur02g00490	BTB/POZ-like
tetur02g00550	Heat_shock_protein_Hsp70
tetur02g00660	phosphoglucose_isomerase
tetur02g00790	MAP_kinase,_p38_n/a_MAP_kinase,_p38_n/a_MAP_kinase,_p38
tetur02g00800	glycogen_debranching_enzyme
tetur02g01290	3-oxoacyl-acyl-carrier-protein_reductase
tetur02g01990	nucleolar_GTP-binding_protein_1_n/a_nucleolar_GTP-binding_protein_1
tetur02g02120	Pyridoxal_phosphate-dependent_transferase,_major_region,_subdomain_1
tetur02g02310	Hypothetical_protein
tetur02g03220	UBA-like
tetur02g03620	Pyridoxal_phosphate-dependent_transferase,_major_region,_subdomain_1
tetur02g03660	BTB/POZ-like
tetur02g03790	CG10920-PA
tetur02g03840	GroEL_like_type_I_chaperonin.
tetur02g03990	Arf_GTPase_activating_protein
tetur02g04250	glutamate_dehydrogenase
tetur02g04270	40S_ribosomal_protein_S2
tetur02g04320	protein_BAT5
tetur02g04560	sumo_ligase
tetur02g04890	Exportin_7
tetur02g05130	cell_differentiation_protein_rcd1
tetur02g05420	WD40/YVTN_repeat-like-containing_domain
tetur02g05550	methylmalonate-semialdehyde_dehydrogenase
tetur02g05880	High_mobility_group_protein_B3,_transcription_factor_protein_n/a_High_mobility_group_protein_B3
tetur02g05900	Protein_kinase-like_domain
tetur02g05980	FERM/acyl-CoA-binding_protein,_3-helical_bundle_n/a_FERM/acyl-CoA-binding_protein,_3-helical_bundle
tetur02g05990	Jumonji/ARID_domain-containing_protein
tetur02g06190	makorin_1
tetur02g06300	multiple_coagulation_factor_deficiency_2
tetur02g06920	Tetratricopeptide_repeat
tetur02g08180	heterogeneous_nuclear_ribonucleoprotein
tetur02g08570	V-type_proton_ATPase_subunit_d_2
tetur02g08890	hypothetical_protein_F42G8.10
tetur02g09420	ubiquitin-protein_ligase_activity
tetur02g09470	Thioredoxin-dependent_peroxide_reductase,_mitochondrial_precursor
tetur02g09480	PRELI_domain-containing_protein_1,_mitochondrial_precursor
tetur02g09610	wings_apart-like_homolog_n/a_wings_apart-like_homolog
tetur02g10920	gamma-soluble_NSF_attachment_protein

tetur02g11130	Peptidase_A22B,_signal_peptide_peptidase
tetur02g11330	Aldo-keto_reductase_family_C_n/a_Hypothetical_protein
tetur02g11430	Manganese/iron_superoxide_dismutase,_C-terminal
tetur02g11480	probable_saccharopine_dehydrogenase
tetur02g11540	Proteasome_subunit_beta_type-2
tetur02g11560	T-complex_protein_1_subunit_gamma
tetur02g11670	muscular_protein_20
tetur02g11760	replication_protein_A_70_kDa_DNA-binding_subunit_n/a_replication_protein_A_70_kDa_DNA-binding_subunit
tetur02g11800	NADH_dehydrogenase,_subunit_TYKY
tetur02g12820	PREDICTED:_similar_to_Dynein_intermediate_chain_2,_cytosolic
tetur02g12910	translocator_protein
tetur02g13310	erlin-1
tetur02g13360	unnamed_protein_product
tetur02g13700	Histidine_phosphatase_superfamily,_clade-1
tetur02g13750	Protein_kinase-like_domain_n/a_Protein_kinase-like_domain
tetur02g14380	Legumain
tetur02g14571	HMG-CoA_synthase_n/a_hypothetical_protein
tetur03g00100	ribosomal_protein_L10
tetur03g00350	proteasome_activator_complex_subunit_3
tetur03g00410	oligoribonuclease
tetur03g00750	Uncharacterised_protein_family_UPF0047
tetur03g01260	Carbohydrate-binding-like_fold
tetur03g01330	paxillin_n/a_paxillin
tetur03g01700	Rho_GDP_dissociation_inhibitor
tetur03g01740	dimethylaniline_monooxygenase
tetur03g01790	gilgamesh_gish_n/a_casein_kinase_I_isoform_gamma-1
tetur03g02660	succinyl-CoA_synthetase_subunit_beta
tetur03g02920	5'-Nucleotidase/apyrase
tetur03g02970	PREDICTED:_PAB-dependent_poly
tetur03g02990	glycogen_synthase_n/a_glycogen_synthase
tetur03g03190	Selenoprotein_15_homologue
tetur03g03220	coatomer_subunit_delta
tetur03g03260	N-alpha-acetyltransferase_35,_NatC_auxiliary_subunit
tetur03g06210	N-acetyltransferase_ARD1_homolog
tetur03g08120	T-complex_protein_1_subunit_beta
tetur03g08530	26S_protease_regulatory_subunit_7
tetur04g01790	catsup_protein
tetur04g01920	mitochondrial_ribosomal_protein_L4_n/a_50S_ribosomal_protein_L4
tetur04g02100	basigin
tetur04g02310	serine_hydroxymethyltransferase
tetur04g02530	Mitochondrial_substrate/solute_carrier
tetur04g02690	Proteasome_subunit_alpha_type-6
tetur04g03120	serine_incorporator_2
tetur04g03250	Peptidyl-prolyl_cis-trans_isomerase,_FKBP-type
tetur04g03850	NSFL1_cofactor_p47
tetur04g03940	Calponin-like_actin-binding
tetur04g04230	elongase,_putative
tetur04g04410	phenylalanine_hydroxylase
tetur04g04770	PREDICTED:_adducin_1_n/a_PREDICTED:_adducin_1
tetur04g04890	isovaleryl-CoA_dehydrogenase
tetur04g05100	WD40/YVTN_repeat-like-containing_domain_n/a_WD40/YVTN_repeat-like-containing_domain
tetur04g05770	sorbitol_dehydrogenase
tetur04g06130	fatty_acid_oxidation_complex_TriFunctional_Enzyme_subunit_alpha,_mitochondrial
tetur04g06230	Y+L_amino_acid_transporter_2
tetur04g06650	Triosephosphate_isomerase
tetur04g06780	mitochondrial_enoyl-CoA_hydratase_n/a_enoyl-CoA_hydratase
tetur04g06990	Cytochrome_bc1_complex,_cytc1_subunit
tetur04g07570	eukaryotic_translation_initiation_factor_3_subunit_D
tetur04g08030	ribosomal_protein_S3
tetur04g08050	serine/threonine-protein_kinase_VRK1
tetur04g08110	elongation_factor_Tu
tetur04g08570	transmembrane_protein_55B
tetur04g08680	Epididymal_secretory_protein_E1_homolog,_Niemann_Pick_type_C2_protein_homolog_n/a_Hypothetical_protein
tetur04g09090	pyruvate_dehydrogenase
tetur05g00290	Rossmann-like_alpha/beta/alpha_sandwich_fold
tetur05g00330	alkaline_phosphatase
tetur05g00750	Proteasome_subunit_alpha_type-3
tetur05g00910	Phosphoribosyl_pyrophosphokinase
tetur05g01580	guanine_nucleotide-binding_protein_G
tetur05g02350	Proliferation-associated_protein_2G4
tetur05g02520	protein_YIPF1
tetur05g02850	Initiation_factor_eIF-4_gamma,_MA3
tetur05g03140	Short-chain_dehydrogenase/reductase_SDR
tetur05g03530	NADH_dehydrogenase
tetur05g03700	myeloid_leukemia_factor_2_n/a_myeloid_leukemia_factor_2
tetur05g03730	transaldolase
tetur05g03950	Uncharacterised_conserved_protein_UCP036436,_nucleotide-sugar_transporter-related
tetur05g04330	WD_repeat_domain_phosphoinositide-interacting_protein_3
tetur05g04670	transitional_endoplasmic_reticulum_ATPase
tetur05g04990	hexokinase
tetur05g06020	sn-1,2-diacylglycerol_ethanolamine_and/or_cholinephosphotranferase
tetur05g06120	Concanavalin_A-like_lectin/glucanase
tetur05g06760	cleavage_stimulation_factor_subunit_2
tetur05g06780	spermidine_synthase
tetur05g06830	proteasomal_ubiquitin_receptor_ADRM1
tetur05g06860	Lupus_La_protein
tetur05g06940	Immunoglobulin_E-set
tetur05g06960	glycosyl_hydrolase_n/a_glycosyl_hydrolase
tetur05g06970	PREDICTED:_Prion-like-
tetur05g07470	Farnesyl_diphosphate_synthase
tetur05g07640	amidase
tetur05g07830	PREDICTED:_similar_to_chromosome_6_open_reading_frame_106
tetur05g08020	aspartyl-tRNA_synthetase
tetur05g08740	putative_enolase_protein
tetur05g08900	ADP-sugar_pyrophosphatase
tetur05g09030	mitochondrial_import_inner_membrane_translocase_subunit_TIM50_precursor
tetur05g09090	T-complex_protein_1_subunit_zeta
tetur05g09170	alpha-actinin-4
tetur05g09385	hypothetical_protein
tetur06g00730	serine_palmitoyltransferase_2
tetur06g00860	Amphiphysin,_Bridging_integrator_1_BIN1_Amphiphysin,Bridging_integrator_1_n/a_amphiphysin
tetur06g01000	cleavage_and_polyadenylation_specificity_factor_subunit_6
tetur06g01370	probable_carboxypeptidase_PM20D1_precursor

tetur06g01780	splicing_factor,_arginine/serine-rich_9
tetur06g01890	regulator_of_chromosome_condensation
tetur06g02230	fatty_acid_synthase
tetur06g02740	Intermediate_filament_protein_n/a_Intermediate_filament_protein
tetur06g02870	RNA-binding_protein_8A
tetur06g02880	homogentisate_1,2-dioxygenase
tetur06g02900	phosphoglucomutase
tetur06g03270	ectonucleotide_pyrophosphatase/phosphodiesterase_family_member_4_precursor
tetur06g03290	ADP/ATP_translocase
tetur06g03940	arg_methyltransferase
tetur06g04370	transcription_initiation_factor_TFIID_subunit_11_n/a_transcription_initiation_factor_TFIID_subunit_11
tetur06g04690	ATP-dependent_metalloprotease_FtsH
tetur06g04850	solute_carrier_family_2,_facilitated_glucose_transporter_member_4
tetur06g05320	Hypothetical_protein
tetur06g05340	transcriptional_repressor_CTCF_zf-C2H2_Zinc_finger,_C2H2_type
tetur06g05840	bifunctional_purine_biosynthesis_protein_PURH
tetur06g05890	H/ACA_ribonucleoprotein_complex_subunit_4
tetur06g05900	unnamed_protein_product
tetur06g06260	stomatin_family_protein
tetur06g06430	26S_proteasome_non-ATPase_regulatory_subunit_12
tetur06g06721	hypothetical_protein
tetur07g00530	Protein_kinase-like_domain
tetur07g00870	Calponin-like_actin-binding
tetur07g01240	ras-related_protein_Rab-5A
tetur07g01490	PREDICTED:_similar_to_protein_phosphatase_1K
tetur07g01530	Histone_H3
tetur07g01990	26S_proteasome_non-ATPase_regulatory_subunit_3
tetur07g02570	Proteasome_subunit_beta_type-1_n/a_Hypothetical_protein
tetur07g02770	60S_ribosomal_protein_L17
tetur07g02820	60S_ribosomal_protein_L7a
tetur07g02960	U5_small_nuclear_ribonucleoprotein_40_kDa_protein
tetur07g03270	Chloride_intracellular_channel_exc-4
tetur07g03440	Fructose-bisphosphate_aldolase,_class-I
tetur07g03460	magnesium-dependent_phosphatase_1
tetur07g03710	ras-related_protein_Rab-8A
tetur07g03840	heat_shock_protein_90
tetur07g04350	PREDICTED:_DnaJ
tetur07g04500	Rpd3,_histone_deacetylase_1_n/a_histone_deacetylase_1
tetur07g04610	AP-2_complex_subunit_alpha-2
tetur07g04660	6-phosphogluconate_dehydrogenase
tetur07g05190	translocation_protein_SEC63_homolog
tetur07g05320	cytochrome_b-c1_complex_subunit_7
tetur07g05380	DnaJ_homolog_subfamily_B_member_11_precursor
tetur07g05480	vitellogenin_fused_with_superoxide_dismutase
tetur07g05720	adenosine_kinase
tetur07g06070	Acyl-CoA_dehydrogenase/oxidase_C-terminal
tetur07g06310	reptin_n/a_ruvB-like_2
tetur07g06340	microtubule_star_mts_n/a_serine/threonine-protein_phosphatase_PP2A_catalytic_subunit
tetur07g06590	Ubiquitin-conjugating_enzyme,_E2
tetur07g06830	serine/threonine-protein_phosphatase_5_n/a_serine/threonine-protein_phosphatase_5
tetur07g06840	manganese-dependent_ADP-ribose/CDP-alcohol_diphosphatase
tetur07g07190	dynamin
tetur07g07260	Protein_kinase-like_domain_n/a_Protein_kinase-like_domain
tetur07g07340	methylenetetrahydrofolate_dehydrogenase
tetur07g07380	beta-galactosidase
tetur07g07520	phosphoglycerate_kinase
tetur07g07530	maf_protein
tetur08g00080	Golgin_subfamily_A_member_7
tetur08g00200	PREDICTED:_similar_to_Activator_of_90_kDa_heat_shock_protein_ATPase_homolog_1
tetur08g00220	Nose_resistant-to-fluoxetine_protein,_N-terminal
tetur08g00430	60S_ribosomal_protein_L11
tetur08g00500	CCR4-NOT_transcription_complex_subunit_7
tetur08g00510	dihydrolipoamide_succinyltransferase_n/a_dihydrolipoamide_succinyltransferase
tetur08g00680	electron_transfer_flavoprotein,_alpha_subunit
tetur08g01260	26S_protease_regulatory_subunit_6A
tetur08g01320	Heat_shock_protein_Hsp70
tetur08g01560	Modifier_of_rudimentary,_putative_transcription_factor_n/a_Modifier_of_rudimentary,_Modr
tetur08g01610	signal_recognition_particle_receptor_subunit_alpha
tetur08g01820	shroud_n/a_corticosteroid_11-beta-dehydrogenase_isozyme_2
tetur08g02020	ubiquitin-conjugating_enzyme_E2_J1
tetur08g02060	3-oxoacyl-acyl-carrier-protein_reductase
tetur08g02080	solute_carrier_family_2,_facilitated_glucose_transporter_member_3
tetur08g02490	UDP-glycosyltransferase,_teturUGT41_UGT_UDP-glycosyltransferase_n/a_glycosyltransferase,_MGT_family
tetur08g02790	Armadillo-type_fold_n/a_Armadillo-type_fold
tetur08g02860	hormone-sensitive_lipase
tetur08g02920	Ras-related_protein_Rap-1b_precursor
tetur08g03210	succinate_dehydrogenase_[ubiquinone]_flavoprotein_subunit_n/a_succinate_dehydrogenase
tetur08g03220	Nascent_polypeptide-associated_complex_subunit_alpha
tetur08g03300	WD_repeat_domain_phosphoinositide-interacting_protein_3
tetur08g03310	membrane-associated_progesterone_receptor_component_2
tetur08g03360	mitogen-activated_protein_kinase_scaffold_protein_1
tetur08g03440	glutamine_synthetase
tetur08g03550	ATP-citrate_synthase
tetur08g03760	EF-hand-like_domain
tetur08g03920	V-type_proton_ATPase_subunit_C_1
tetur08g03990	Coatomer_subunit_epsilon_n/a_Coatomer_subunit_epsilon
tetur08g04930	eukaryotic_translation_initiation_factor_4E_nuclear_import_factor_1_n/a_eukaryotic_translation_initiation_factor_4E_nuclear_import_factor_1
tetur08g05040	downstream_of_receptor_kinase;_growth_factor_receptor-bound_protein_2_n/a_growth_factor_receptor-bound_protein_2
tetur08g05320	disintegrin_and_metalloproteinase_domain-containing_protein_10
tetur08g05380	PREDICTED:_similar_to_AGAP007375-PA
tetur08g05630	60S_ribosomal_protein_L9
tetur08g05730	PREDICTED:_solute_carrier_family_7
tetur08g06180	Cleavage_and_polyadenylation_specificity_factor_subunit_5
tetur08g06190	26S_protease_regulatory_subunit_8
tetur08g06510	Trafficking_protein_particle_complex_subunit_3
tetur08g06560	pentatricopeptide_repeat-containing_protein_2
tetur08g06680	coiled-coil_domain-containing_protein_25

tetur08g07120	glutaredoxin
tetur08g07310	PREDICTED:_similar_to_adenylate_kinase_3
tetur08g07360	peptidyl-prolyl_cis-trans_isomerase_FKBP4
tetur08g07400	C-1-tetrahydrofolate_synthase,_cytoplasmic
tetur08g07540	solute_carrier_family_25,_member_1
tetur08g08339	Zinc_finger_protein_ZPR1_n/a_hypothetical_protein
tetur09g00340	uridine_5'-monophosphate_synthase
tetur09g00390	Regulator_of_nonsense_transcripts_n/a_ATP-dependent_helicase_NAM7
tetur09g01460	PREDICTED:_similar_to_haloacid_dehalogenase-like_hydrolase_domain_containing_1A
tetur09g01620	ADP-ribosylation_factor-like_protein_5A
tetur09g01650	UDP-glycosyltransferase,_teturUGT46_UGT_UDP-glycosyltransferase_n/a_Hypothetical_protein
tetur09g01720	aminopeptidase
tetur09g02070	Armadillo-type_fold
tetur09g02080	Zinc_finger,_MIZ-type
tetur09g03350	tropomyosin
tetur09g03760	secreted_salivary_gland_protein,_putative_n/a_secreted_salivary_gland_peptide,_putative
tetur09g03850	Casein_kinase_II_subunit_alpha
tetur09g04020	synaptobrevin_homolog_YKT6
tetur09g04090	eukaryotic_translation_initiation_factor_2_subunit_1_n/a_eukaryotic_translation_initiation_factor_2_subunit_1
tetur09g04160	phosphatidylserine_synthase_1
tetur09g04800	Gamma-interferon-inducible_lysosomal_thiol_reductase_precursor_n/a_Gamma-interferon-inducible_lysosomal_thiol_reductase_precursor
tetur09g04810	Eukaryotic_translation_initiation_factor_1A,_X-chromosomal
tetur09g04820	low_density_lipoprotein_receptor_adapter_protein_1
tetur09g05120	WD_repeat_and_FYVE_domain-containing_protein_2_n/a_WD_repeat_and_FYVE_domain-containing_protein_2
tetur09g05150	endoplasmin
tetur09g05440	lipoic_acid_synthetase
tetur09g05730	K_Homology,_type_1
tetur09g06290	glycerol-3-phosphate_dehydrogenase
tetur09g06350	isocitrate_dehydrogenase_1,_putative_mitochondrial_isoform_n/a_isocitrate_dehydrogenase_1
tetur09g06530	microspherule_protein_1
tetur101g00020	NEDD8-conjugating_enzyme_Ubc12
tetur10g00450	Proteasome_beta-5_subunit_n/a_PREDICTED:_similar_to_proteasome
tetur10g00490	2-oxoisovalerate_dehydrogenase_subunit_beta,_mitochondrial_precursor
tetur10g00800	cell_cycle_associated_protein_1
tetur10g00980	Phox-like
tetur10g01140	ornithine_decarboxylase_n/a_ornithine_decarboxylase
tetur10g01370	HLA-B_associated_transcript_3
tetur10g02330	cysteine_desulfurase
tetur10g02620	Myosin_regulatory_light_chain_2,_smooth_muscle_isoform
tetur10g02780	serine_carboxypeptidase,_putative
tetur10g02790	S-formylglutathione_hydrolase
tetur10g03230	dihydrolipoamide_dehydrogenase
tetur10g03400	Mob1/phocein
tetur10g03840	dolichyl-diphosphooligosaccharide--protein_glycosyltransferase_48_kDa_subunit_precursor
tetur10g04120	seryl-tRNA_synthetase
tetur10g04340	NADH_cytochrome_b5_reductase_gene_b
tetur10g04380	Guanylate-binding_protein,_C-terminal
tetur10g04570	GOLD
tetur10g04830	geranylgeranyl_pyrophosphate_synthase
tetur10g05140	D-isomer_specific_2-hydroxyacid_dehydrogenase_family_protein
tetur10g05150	WD40_repeat-containing_protein_n/a_Hypothetical_protein
tetur11g00350	6-phosphofructo-2-kinase/fructose-2,6-biphosphatase_1
tetur11g00580	Ubiquitin-conjugating_enzyme,_E2_n/a_Ubiquitin-conjugating_enzyme,_E2
tetur11g01320	electron_transfer_flavoprotein,_beta_subunit
tetur11g01700	Heat_shock_protein_Hsp70
tetur11g02240	calmodulin
tetur11g02780	pyruvate_dehydrogenase_complex_dihydrolipoamide_acetyltransferase
tetur11g03050	phosphoserine_phosphatase_n/a_phosphoserine_phosphatase
tetur11g03290	ATP_synthase_subunit_delta,_mitochondrial_precursor
tetur11g03320	TBC1_domain_family_member_7
tetur11g03360	NADH-quinone_oxidoreductase,_49kDA_subunit
tetur11g03370	GOLD
tetur11g03550	thioredoxin
tetur11g03700	JH_epoxide_hydrolase
tetur11g03980	Nucleotide-binding,_alpha-beta_plait
tetur11g04230	Phosphoribosyl_pyrophosphokinase
tetur11g04560	26S_protease_regulatory_subunit_S10B
tetur11g04630	Aph-1_n/a_Aph-1
tetur11g05620	Ubiquinone_biosynthesis_protein_COQ7_homolog
tetur11g05670	ornithine_aminotransferase
tetur11g06372	Sorbitol_dehydrogenase_n/a_n/a
tetur11g06430	hypothetical_protein
tetur12g00070	phosphoenolpyruvate_carboxykinase
tetur12g00330	mitochondrial_ribosomal_protein_L39
tetur12g00850	Vigilin,_High_density_lipoprotein-binding_protein_n/a_vigilin
tetur12g01490	sorting_nexin-6
tetur12g01800	ubiquitin-activating_enzyme_E1
tetur12g01840	Cathepsin_L_n/a_proteinase_inhibitor,_cathepsin_propeptide_n/a_Hypothetical_protein
tetur12g01860	Cathepsin_L
tetur12g01960	Protein_phosphatase_2A_at_29B_Pp2A-29B_n/a_protein_phosphatase_2
tetur12g02190	PREDICTED:_DEAD
tetur12g02480	Concanavalin_A-like_lectin/glucanase
tetur12g03460	YIP1_trans-membrane_protein_from_the_Golgi_apparatus
tetur12g03490	Protein_tyrosine_phosphatase-like_protein
tetur12g03640	conserved_hypothetical_protein
tetur12g03690	6-phosphofructokinase,_muscle_type
tetur12g03790	2,3-bisphosphoglycerate-independent_phosphoglycerate_mutase
tetur12g04280	SecY_protein
tetur12g04290	cytosolic_malate_dehydrogenase
tetur13g00130	pyruvate_kinase_n/a_pyruvate_kinase
tetur13g00240	lysyl-tRNA_synthetase
tetur13g01190	Aconitase/3-isopropylmalate_dehydratase_large_subunit,_alpha/beta/alpha,_subdomain_1/3
tetur13g01410	DnaJ
tetur13g01820	PREDICTED:_ubiquitin_specific_peptidase_5
tetur13g01840	AP-1_complex_subunit_mu-1
tetur13g02070	2-amino-3-ketobutyrate_coenzyme_A_ligase
tetur13g02120	ER_degradation-enhancing_alpha-mannosidase-like_1
tetur13g02480	capping_protein
tetur13g02500	Hypothetical_protein
tetur13g02710	Protein_kinase-like_domain

tetur13g03230	Translation_elongation/initiation_factor/Ribosomal,_beta-barrel
tetur13g03380	protein_YIF1A
tetur13g03690	vismay_n/a_TGFB-induced_factor_homeobox_1
tetur13g03860	T-complex_protein_1_subunit_eta
tetur13g04000	Cell_division_control_protein_42_homolog_precursor
tetur13g04270	transmembrane_protein_120A
tetur13g04330	innexin,_putative
tetur14g00600	casein_kinase_II_beta_subunit_n/a_casein_kinase_II_beta_subunit
tetur14g00690	Adenylate_cyclase-associated_CAP
tetur14g00730	elongation_factor-2
tetur14g00900	UBA-like
tetur14g01630	B-cell_receptor-associated_protein_31
tetur14g01800	translationally_controlled_tumor_protein
tetur14g02040	Phox-like
tetur14g02400	Hypothetical_protein
tetur14g02430	6-phosphogluconolactonase
tetur14g02500	Hypothetical_protein
tetur14g02670	Ras_small_GTPase,_Rab_type
tetur14g03140	Protein_Disulfide_Isomerase_n/a_Hypothetical_protein
tetur14g03190	hypothetical_protein_IscW_ISCW022785
tetur14g03410	Zinc_finger,_RING-CH-type
tetur14g03490	Hypothetical_protein
tetur14g03530	Cytochrome_bc1_complex,_Rieske_protein
tetur14g03700	cyclin-dependent_kinase_4
tetur14g03740	guanine_nucleotide_binding_protein_subunit_beta-like_RACK1_guanine_nucleotide_binding_protein_subunit_beta-like_n/a_guanine_nucleotide_binding_protein
tetur15g00380	PREDICTED:_similar_to_CG9380_CG9380-PB
tetur15g00400	Zinc_finger,_RING-type
tetur15g00550	PREDICTED:_similar_to_F55A4.8a
tetur15g00900	flotillin_2
tetur15g01880	Similar_to_Autophagy-specific_gene_8a_n/a_Gamma-aminobutyric_acid_receptor-associated_protein
tetur15g01920	Ras_small_GTPase
tetur15g02350	Ubiquitin_fusion_degradation_protein_1_homolog
tetur15g02790	PREDICTED:_similar_to_Chromosome_2_open_reading_frame_13_n/a_PREDICTED:_similar_to_Chromosome_2_open_reading_frame_13
tetur15g03240	glucose-6-phosphate_1-dehydrogenase
tetur15g03340	Nonaspanin_TM9SF
tetur15g03360	Serine/threonine-specific_protein_phosphatase/bis5-nucleosyl-tetraphosphatase
tetur15g03750	PREDICTED:_similar_to_solute_carrier_family_20
tetur16g00250	scaffold_protein
tetur16g00540	Eukaryotic_peptide_chain_release_factor_eRF_subunit-1
tetur16g00560	ribosomal_protein_L26
tetur16g00580	26S_proteasome_non-ATPase_regulatory_subunit_4
tetur16g01280	Endonuclease/exonuclease/phosphatase
tetur16g01440	inorganic_pyrophosphatase
tetur16g01490	Rab_geranylgeranyltransferase,_beta_subunit
tetur16g01940	nucleolar_protein_56
tetur16g02150	hypothetical_protein_n/a_hypothetical_protein
tetur16g02500	1,4-alpha-glucan_branching_enzyme
tetur16g02710	mitochondrial_carnitine/acylcarnitine_carrier_protein
tetur16g03110	eukaryotic_translation_initiation_factor_3_subunit_H
tetur17g01550	protein_lin-7_homolog_B
tetur17g01680	TGF-beta-inducible_nuclear_protein_1
tetur17g01700	GF11309
tetur17g01780	3-hydroxyisobutyrate_dehydrogenase
tetur17g02020	ubiquitin_conjugating_enzyme
tetur17g02060	secreted_salivary_gland_peptide,_putative_n/a_secreted_salivary_gland_peptide,_putative
tetur17g02070	sirtuin_1_n/a_sirtuin_1
tetur17g02100	H/ACA_ribonucleoprotein_complex_subunit_2-like_protein
tetur17g02600	downstream_of_raf1_n/a_dual_specificity_mitogen-activated_protein_kinase_kinase_1
tetur17g02880	Peptidase_M16,_core
tetur17g03110	signal_recognition_particle_54_kDa_protein_n/a_signal_recognition_particle_54_kDa_protein
tetur17g03570	innexin,_putative
tetur17g03710	innexin,_putative
tetur17g04282	hypothetical_protein
tetur18g00250	T-complex_protein_1_subunit_alpha
tetur18g00610	dihydropyrimidinase-related_protein_3
tetur18g00950	PREDICTED:_similar_to_Y4C6B.2a
tetur18g01060	mitochondrial_malate_dehydrogenase_2,_NAD
tetur18g01200	PREDICTED:_similar_to_ubiquitin-conjugating_enzyme_E2W
tetur18g01720	myosin-10
tetur18g01910	hypothetical_protein_Kpol_483p11
tetur18g02350	PREDICTED:_achalasia,_adrenocortical_insufficiency,_alacrimia
tetur18g02380	ataxin-2
tetur18g02560	protein_kinase_C_alpha_type
tetur18g03580	malate_dehydrogenase
tetur19g00170	26S_proteasome_non-ATPase_regulatory_subunit_8
tetur19g00560	LisH_dimerisation_motif
tetur19g00580	syntaxin-7
tetur19g00610	26S_proteasome_non-ATPase_regulatory_subunit_14
tetur19g01020	sphingolipid_delta
tetur19g01670	motile_sperm_domain_containing_2
tetur19g01850	lipase_maturation_factor_2_n/a_lipase_maturation_factor_2
tetur19g01900	PREDICTED:_similar_to_prolylcarboxypeptidase
tetur19g01980	Fumarate_hydratase,_probably_mitochondrial
tetur19g02170	ferritin
tetur19g03090	uroporphyrinogen_decarboxylase
tetur19g03100	PREDICTED:_similar_to_CCR4-NOT_transcription_complex_subunit_2_n/a_PREDICTED:_similar_to_CCR4-NOT_transcription_complex_subunit_2
tetur20g01500	Uncharacterised_protein_family_UPF0224
tetur20g01760	proliferating_cell_nuclear_antigen
tetur20g01920	Chaperonin_Cpn60
tetur20g02640	PREDICTED:_similar_to_Fasciclin-1_precursor
tetur20g02910	ortholog_of_Argonaute-1_Drosophila_melanogaster_AGO-1A_translation_initiation_factor_AGO-1A_ortholog_of_Dm_Argonaute-1
tetur20g03050	Vacuolar_proton_pump_subunit_D
tetur20g03120	Transmembrane_emp24_domain-containing_protein_5_precursor
tetur20g03160	PREDICTED:_similar_to_vitelline_membrane_outer_layer_1_homolog
tetur20g03250	Carboxyl/cholinesterase_TuCCE-42_Carboxyl/cholinesterase
tetur21g00060	lethal
tetur21g00570	translocon-associated_protein_subunit_alpha
tetur21g00980	roundabout,_axon_guidance_receptor,_homolog_1_n/a_roundabout,_axon_guidance_receptor,_homolog_1
tetur21g01050	PREDICTED:_similar_to_methylcrotonoyl-Coenzyme_A_carboxylase_2
tetur21g01450	Armadillo-type_fold
tetur21g01860	PREDICTED:_similar_to_poly
tetur21g01970	hexokinase
tetur21g02180	protein_LSM14_homolog_A

tetur21g02310	TWINS,_Protein_phosphatase_PP2A_55_kDa_regulatory_subunit_B
tetur21g02410	lysophospholipase
tetur21g02490	methylenetetrahydrofolate_dehydrogenase
tetur21g02560	inactive_hydroxysteroid_dehydrogenase-like_protein_1
tetur224g00010	PREDICTED:_similar_to_glucose_dehydrogenase
tetur22g00230	ribosome_biogenesis_protein_BRX1_homolog
tetur22g00900	ectonucleoside_triphosphate_diphosphohydrolase_n/a_ectonucleoside_triphosphate_diphosphohydrolase_6
tetur22g01290	CDP-diacylglycerol--inositol_3-phosphatidyltransferase
tetur22g01510	replication_factor_C_subunit_4
tetur22g02560	NADH-quinone_oxidoreductase,_51_kDa_subunit
tetur22g02630	proteasome
tetur23g00430	eukaryotic_translation_initiation_factor_3_subunit_E
tetur23g00710	cystathionine_gamma-lyase
tetur23g00720	T-complex_protein_1_subunit_delta
tetur23g01110	UDP-N-acetylglucosamine_pyrophosphorylase
tetur23g01290	Cathepsin_L
tetur23g01840	CG10719
tetur23g01950	High_mobility_group,_HMG1/HMG2_n/a_High_mobility_group,_HMG1/HMG2
tetur23g02420	cell_division_cycle_protein_20_homolog_n/a_cell_division_cycle_protein_20_homolog
tetur23g02451	Glycerol-3-phosphate_dehydrogenase,_mitochondrial_n/a_n/a
tetur24g01160	Mov34/MPN/PAD-1
tetur24g01190	Thiolase-like,_subgroup
tetur24g01600	40S_ribosomal_protein_S4
tetur24g01750	protein_phosphatase_1A
tetur24g01930	lysosomal_alpha-mannosidase
tetur24g01980	short-chain_dehydrogenase/reductase_SDR_n/a_short-chain_dehydrogenase/reductase_SDR
tetur24g02190	U2_auxiliary_factor_small_subunit
tetur25g00020	Zinc_finger,_Sec23/Sec24-type
tetur25g00240	Adhesion_molecule_CD36
tetur25g00250	Glyceraldehyde-3-phosphate_dehydrogenase
tetur25g00370	DNA_breaking-rejoining_enzyme,_catalytic_core
tetur25g00440	Neuroendocrine_7B2_precursor
tetur25g00690	acyl-CoA_dehydrogenase
tetur25g00840	Ovarian_tumour,_otubain
tetur25g01400	actin-related_protein_2
tetur25g01910	cytoplasmic_Threonyl-tRNA_synthetase,_putative_n/a_tRNA_synthetase_n/a_unnamed_protein_product
tetur26g00240	ras-related_protein_Rab-7a_n/a_ras-related_protein_Rab-7a
tetur26g00720	ribosome_production_factor_1;_U3_Small_nucleolar_ribonucleoprotein_IMP4_RPF1_ribosome_production_factor_1
tetur26g00940	eukaryotic_translation_initiation_factor_3_subunit_M
tetur26g01690	numb_homolog_n/a_numb_homolog
tetur26g02130	Transmembrane_emp24_domain-containing_protein_10_precursor
tetur26g02150	sphingomyelin_phosphodiesterase_n/a_sphingomyelin_phosphodiesterase
tetur26g02340	WD40/YVTN_repeat-like-containing_domain
tetur26g02480	alcohol_dehydrogenase_class_3
tetur27g00690	scavenger_receptor_activity
tetur27g00800	calnexin
tetur27g01090	tyrosine-protein_phosphatase_non-receptor_type_4
tetur27g01310	Vitellogenin_receptor_Vitellogenin_rec_Vitellogenin_receptor
tetur27g01330	Mid1-interacting_protein_1
tetur27g01540	Transportin_1
tetur27g01760	transcription_initiation_factor_IIB
tetur27g02140	aldehyde_dehydrogenase
tetur27g02190	asparaginyl-tRNA_synthetase
tetur27g02390	THO_complex_subunit_7_homolog_n/a_THO_complex_subunit_7_homolog
tetur28g00190	metalloproteinase_inhibitor_3_precursor
tetur28g00290	Zinc_finger,_PHD-type
tetur28g00490	Estrogen-related_Receptor
tetur28g00520	phospholipid_hydroperoxide_glutathione_peroxidase
tetur28g00710	secreted_salivary_gland_peptide,_putative
tetur28g00760	integral_membrane_protein_2B
tetur28g00850	unnamed_protein_product
tetur28g00890	coatomer_subunit_gamma-2
tetur28g01500	yippee-like_5
tetur28g01520	Programmed_cell_death_protein_6
tetur28g01550	SPRY_domain-containing_SOCS_box_protein_1
tetur28g01610	transcription_factor_E2F3
tetur28g01920	60S_ribosomal_protein_L13a
tetur28g02090	Zinc_finger,_B-box
tetur28g02200	polyadenylate-binding_protein_2
tetur28g02400	60S_ribosomal_protein_L10a
tetur29g00250	stress-induced-phosphoprotein_1
tetur29g00620	ABC-transporter,_class_F
tetur29g00840	NADH_dehydrogenase
tetur29g01040	histone-binding_protein_RBBP7
tetur29g01140	DNA-directed_RNA_polymerases_I,_II,_and_III_subunit_RPABC1_n/a_DNA-directed_RNA_polymerases_I,_II,_and_III_subunit_RPABC1
tetur29g01180	Carbonic_anhydrase_2
tetur29g01230	Levanase,_Beta-D-fructofuranosidase,_secreted_n/a_levanase
tetur29g01530	unnamed_protein_product
tetur29g01600	Ras_small_GTPase,_Rab_type
tetur29g01640	glycogenin
tetur30g00130	sorting_nexin_3
tetur30g00270	transcription_factor_AP-4_activating_enhancer_binding_protein_4
tetur30g00470	ribosomal_protein_L4
tetur30g00510	Nuclear_transcription_factor_Y_subunit_beta_n/a_nuclear_transcription_factor_Y_subunit_beta
tetur30g00940	vacuolar_protein-sorting-associated_protein_25
tetur30g01020	Plexin-like_fold
tetur30g01620	tropomodulin_n/a_tropomodulin
tetur30g02190	Possible_homolog_of_the_DHR78_NR2D1_NR2C-like_NR2C/D-like_nuclear_receptor
tetur30g02220	Ubiquitin_supergroup
tetur31g01010	Ras-induced_vulval_development_antagonist,_synthetic_multivulva_synMuv_homolog._synMuvL_Ras-induced_vulval_development_antagonist,_synthetic_multivulva_synMuv_homolog._n/a_Protein_of_unknown_function_DUF926
tetur31g01130	sphingosine-1-phosphate_lyase
tetur31g01250	ethanolamine-phosphate_cytidylyltransferase
tetur31g01350	synaptosomal-associated_protein_29
tetur31g01360	synapse-associated_protein_1
tetur31g01630	ester_hydrolase_C11orf54_homolog
tetur31g01800	ribosomal_protein_S5
tetur32g00040	LIM_domain_family_member_n/a_LIM_domain_family_member
tetur32g00130	3-hydroxyisobutyryl-CoA_hydrolase,_mitochondrial_precursor
tetur32g00170	Signal_peptidase_complex_catalytic_subunit_SEC11A_n/a_Signal_peptidase_complex_catalytic_subunit_SEC11A
tetur32g00200	myosin_alkali_light_chain
tetur32g00390	jagunal
tetur32g00400	inositol_monophosphatase_1
tetur32g00520	eukaryotic_translation_initiation_factor_3_subunit_C

tetur32g00630	ribosomal_protein_L6
tetur32g01370	oxysterol_binding_protein-like_9
tetur32g01380	Pepsin
tetur32g01790	adenosine_kinase
tetur32g01800	26S_protease_regulatory_subunit_6B
tetur32g01840	PREDICTED:_similar_to_DnaJ
tetur32g02010	40S_ribosomal_protein_SA
tetur32g02020	amino_acid_transporter
tetur33g00180	piperideine-6-carboxylate_dehydrogenase
tetur33g00210	glucose-6-phosphatase
tetur33g00280	CG4538-PA
tetur33g00770	Thioredoxin-like_fold
tetur33g00810	alcohol_dehydrogenase
tetur33g01390	26S_proteasome_non-ATPase_regulatory_subunit_6
tetur34g00490	DJ-1_family_protein
tetur34g00590	Insulin-like_growth_factor-binding_protein,_IGFBP
tetur34g01000	mago_nashi_n/a_mago_nashi
tetur34g01130	quaking_protein
tetur35g00170	mitochondrial_import_receptor_subunit_TOM40_homolog
tetur35g00260	translation_initiation_factor_2_gamma_subunit
tetur35g00610	TBC1_domain_family_member_20
tetur35g00630	COX11_homolog,_cytochrome_c_oxidase_assembly_protein
tetur35g00670	rab_GDP_dissociation_inhibitor_alpha
tetur35g00730	Sodium/potassium-transporting_ATPase_subunit_beta_Nrv_cation_transmembrane_transporter_activity
tetur35g00760	26S_protease_regulatory_subunit_4
tetur36g00130	Ribosomal_protein_S24
tetur36g00330	NADH_dehydrogenase
tetur36g00480	transcription_factor_Dp-1
tetur36g00780	cytochrome_c_oxidase_polypeptide_Vb
tetur36g00920	Cytochrome_P450_-_CYP391A1
tetur37g00130	conserved_edge_expressed_protein
tetur37g00840	Cyclin-like
tetur37g00950	autophagy-related_protein_3
tetur38g00110	protein_BTG3
tetur38g00200	Phosphotyrosine_interaction_domain
tetur38g00240	collagen_alpha-2
tetur38g00420	peptidylprolyl_isomerase_B
tetur39g00490	Programmed_cell_death_protein_6
tetur39g00510	COP9_signalosome_complex_subunit_5
tetur39g00670	calcyphosin-like_protein
tetur40g00210	Hypothetical_protein
tetur40g00230	ATP_synthase_subunit_b,_mitochondrial_precursor
tetur41g00210	ATP_synthase_beta_subunit
tetur41g00250	transmembrane_protein_33
tetur41g00530	Vacuolar_proton_pump_subunit_E_1_n/a_Vacuolar_proton_pump_subunit_E_1
tetur43g00240	Clathrin/coatomer_adaptor,_adaptin-like,_appendage,_Ig-like_subdomain
tetur43g00290	Transmembrane_receptor,_eukaryota
tetur47g00180	NADH-ubiquinone_oxidoreductase_75_kDa_subunit,_mitochondrial_precursor

*Gene ID is according to the coding DNA sequence (CDS) of *Tetranychus urticae* [18].
