# Supplementary figures and images for: Phylogeny of the spider mite sub-family Tetranychinae (Acari: Tetranychidae) inferred from RNA-Seq data
Source: PLoS One. 2018 Sep 7;13(9):e0203136. doi: 10.1371/journal.pone.0203136 (PMC6128517; doi:10.1371/journal.pone.0203136)

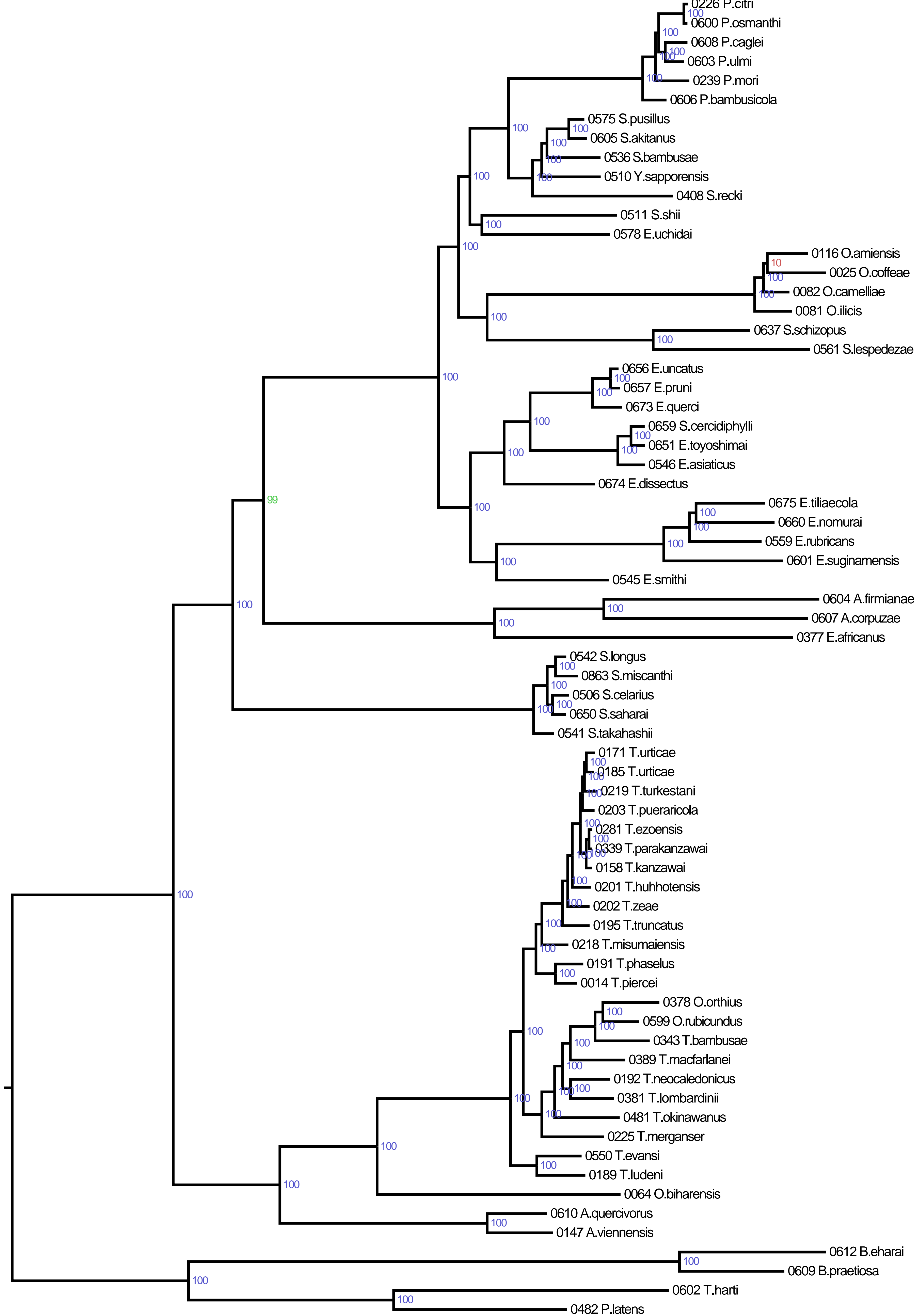

Supplement: S1 Fig — (PDF) [file pone.0203136.s003.pdf]
